# Metabolic modulation of melanoma enhances the therapeutic potential of immune checkpoint inhibitors

**DOI:** 10.3389/fonc.2024.1428802

**Published:** 2024-10-01

**Authors:** Zafer Gurel, Michael S. Luy, Qianyun Luo, Nicholas L. Arp, Amy K. Erbe, Aparna H. Kesarwala, Jing Fan, Randall J. Kimple

**Affiliations:** ^1^ Department of Human Oncology, University of Wisconsin School of Medicine and Public Health, Madison, WI, United States; ^2^ Morgridge Institute for Research, Madison, WI, United States; ^3^ Department of Radiation Oncology, Winship Cancer Institute, Emory University School of Medicine, Atlanta, GA, United States; ^4^ Cellular and Molecular Biology Graduate Program, University of Wisconsin-Madison, Madison, WI, United States; ^5^ Department of Nutritional Sciences, University of Wisconsin-Madison, Madison, WI, United States; ^6^ University of Wisconsin (UW) Carbone Cancer Center, University of Wisconsin School of Medicine and Public Health, Madison, WI, United States

**Keywords:** melanoma, cancer metabolism, lactate, LDH, oxamate, NHI-2, immune checkpoint inhibitors

## Abstract

**Introduction:**

Lactate is a pivotal molecule with diverse functions in the metabolic reprogramming of cancer cells. Beyond its role in metabolism, lactate exerts a modulatory effect within the tumor microenvironment; it is utilized by stromal cells and has been implicated in the suppression of the immune response against the tumor.

**Methods:**

Using *in vitro* assays (including flow cytometry, live-cell imaging and metabolic analyses), the impact of lactate dehydrogenase inhibitors (LDHIs) on melanoma cells were assessed. The therapeutic potential of LDHIs with immune checkpoint inhibitors (ICIs) were tested *in vivo* in murine models of melanoma tumors.

**Results:**

A potent anti-proliferative effect (via both cell cycle alterations and enhanced apoptosis) of LDHIs, Oxamate (Oxa) and methyl 1-hydroxy-6-phenyl-4-(trifluoromethyl)-1H-indole-2-carboxylate (NHI-2), was found upon treatment of melanoma cell lines. Using a combination of Oxa and NHI-2, a synergistic effect to inhibit proliferation, glycolysis, and ATP production was observed. Metabolic analysis revealed significant alteration in glycolysis and oxidative phosphorylation, while metabolite profiling emphasized consequential effects on lactate metabolism and induced energy depletion by LDHIs. Detection of increased RANTES and MCP-1, with Oxa and NHI-2 treatment, prompted the consideration of combining LDHIs with ICIs. *In vivo* studies using a murine B78 melanoma tumor model revealed a significant improvement in treatment efficacy when LDHIs were combined with ICIs.

**Conclusions:**

These findings propose the potential of targeting lactate metabolism to enhance the efficacy of ICI treatments in patients with melanoma.

## 1 Introduction

The metabolism of cancer cells differs markedly from that of non-malignant cells. One of the major metabolic hallmarks of cancer cells, the Warburg effect, was reported almost a century ago (1). The Warburg effect is defined as an increased dependence on glycolysis for ATP synthesis, even in the presence of oxygen, diverging from the conventional oxidative phosphorylation pathway (2). Subsequent studies have revealed that the metabolic adaptations in tumors extend beyond the Warburg effect (3). Cancer cells demonstrate notable metabolic plasticity, enabling them to swiftly adjust to the dynamically changing tumor microenvironment (TME) (4). This metabolic plasticity, coupled with genetic and epigenetic alterations contributes to heterogeneity within the tumor, resulting in chemo/radio-resistance, immune escape, and tumor recurrence (5–7).

Lactate is a metabolic byproduct that has long been considered a waste product of glycolysis. Although the initial reports of lactate accumulation in muscles date back to the early 1900s (8, 9), it is only within the last two decades that we have started to unravel novel biological functions associated with this molecule. Lactate is now recognized as both an important carbon source for cellular metabolism and as a signaling molecule, particularly in chronically inflamed and cancerous tissues (10). However, the cellular response to lactate in the TME is quite different to that occurring in the context of chronic inflammation. Therefore, further studies are required to understand the role of lactate in the TME.

Given the metabolic diversity among tumor cells, there is a postulation that glycolytic and oxidative cells engage in symbiotic interactions. Glycolytic cells export lactate to the TME, while oxidative cells import it and utilize lactate as an energy source (11). This symbiotic relationship becomes particularly crucial as tumors grow, leading to a hypoxic microenvironment in the tumor center and better oxygenation near blood vessels (12). The utilization of lactate as an energy source by perivascular cells in tumors could elucidate the correlation between elevated lactate concentrations in the TME and the subsequent development of nodal or distant metastases in various cancers (13–19).

Moreover, high TME lactate levels play a crucial role in modulating immune cell function and fostering immune escape within tumors by suppressing the tumor-specific CD8^+^ T lymphocytes and macrophages (20–22). Additionally, lactate-driven immune cell modulation includes an influx of suppressive immune subsets, with Treg cells adapting to the high-lactate/low-glucose environment by upregulating FOXP3 expression (23). This multilayered impact of lactate highlights its significance in tumor progression and immune response modulation.

In the landscape of melanoma treatment, immune checkpoint inhibitors (ICI) have emerged as a cornerstone over the past decade, revolutionizing therapeutic strategies for many patients. However, both innate and acquired resistance to ICI treatment present substantial challenges, limiting the therapeutic impact of ICI therapy in melanoma. Several checkpoint inhibitors, including the anti-CTLA-4 and the anti-PD-1 monoclonal antibodies, are approved for the treatment of advanced melanoma. Studies dedicated to melanoma treatment report response rates of 20% for anti-CTLA-4 and 40% for anti-PD-1, accompanied by 5-year progression-free survival (PFS) rates of 8% and 20%, respectively (24–27). These insights highlight the existing challenges and emphasize the need for innovative approaches to enhance the efficacy of ICI treatments in melanoma. Several clinical trials are in progress to evaluate the efficacy of combining glycolysis inhibitors with immune modulator therapies, predominantly focusing on the AKT/mTOR pathway (NCT03190174, NCT03772561, NCT04895748, NCT04591431). However, none of these clinical trials focus on melanoma patients and there are only a limited number of pre-clinical studies that utilize lactate dehydrogenase inhibitors (LDHIs) with ICIs in animal tumor models (28, 29).

Lactate dehydrogenase (LDH) is a critical enzyme in the metabolic process, serving a pivotal role in the interconversion of lactate and pyruvate, alongside the concomitant interconversion of NADH and NAD+ (30). LDH comprises multiple isoenzymes, among which LDHA and LDHB are the primary subunits that combine to form the various LDH isoforms present in human tissues (31). These subunits are encoded by distinct genes, LDHA and LDHB, respectively, and their expression and activity are finely tuned according to the metabolic demands and oxygen availability of specific tissues. The link between elevated serum LDH levels and poor prognosis in melanoma is well established, indicating the significance of LDH as a prognostic biomarker (32–35). Additionally, high baseline LDH levels and lactic acid accumulation have been correlated with less favorable outcomes in patients receiving ICIs, indicating that targeting LDH could enhance the efficacy of ICI therapies in melanoma (36–39).

This study aimed to disrupt the energy metabolism within melanoma tumors by targeting LDH enzymes, specifically LDHA and LDHB, with the goal of enhancing the efficacy of ICI treatments. We observed strong anti-proliferative effects of two LDHIs, namely Oxamate (Oxa) and Methyl 1-hydroxy-6-phenyl-4-(trifluoromethyl)-1H-indole-2-carboxylate (NHI-2), on melanoma cell lines. Oxa, acting as a pyruvate analog inhibits LDHA (40), whereas NHI-2 functions as a NADH competitor to inhibit both LDHA and LDHB (41). The observed synergistic interaction between Oxa and NHI-2 revealed an impact on melanoma cell metabolism that extends beyond glycolysis inhibition, including oxidative phosphorylation and macromolecule synthesis. These findings point to a more complex mechanism of action, underscoring the potential significance of this drug combination in targeting melanoma cells. Additionally, screening for changes in cytokines and chemokines showed an elevation in the release of specific chemokines, including regulated upon activation, normal T cell expressed and secreted (RANTES), and monocyte chemoattractant protein-1 (MCP-1) in the B78 melanoma cell line following LDHI treatment. *In vivo* studies showed a significant improvement in treatment efficacy when Oxa and NHI-2 were combined with immune checkpoint inhibitors (aPD-L1+aCTL4) in a B78 melanoma tumor model. Therefore, this study not only indicated the potential strategy of disrupting melanoma cell metabolism through LDHIs but also emphasized the innovative approach of enhancing ICI efficacy through this combination.

## 2 Results

### 2.1 Synergistic anti-proliferative effects of LDH inhibitors on melanoma cell lines

**Figure 1:**
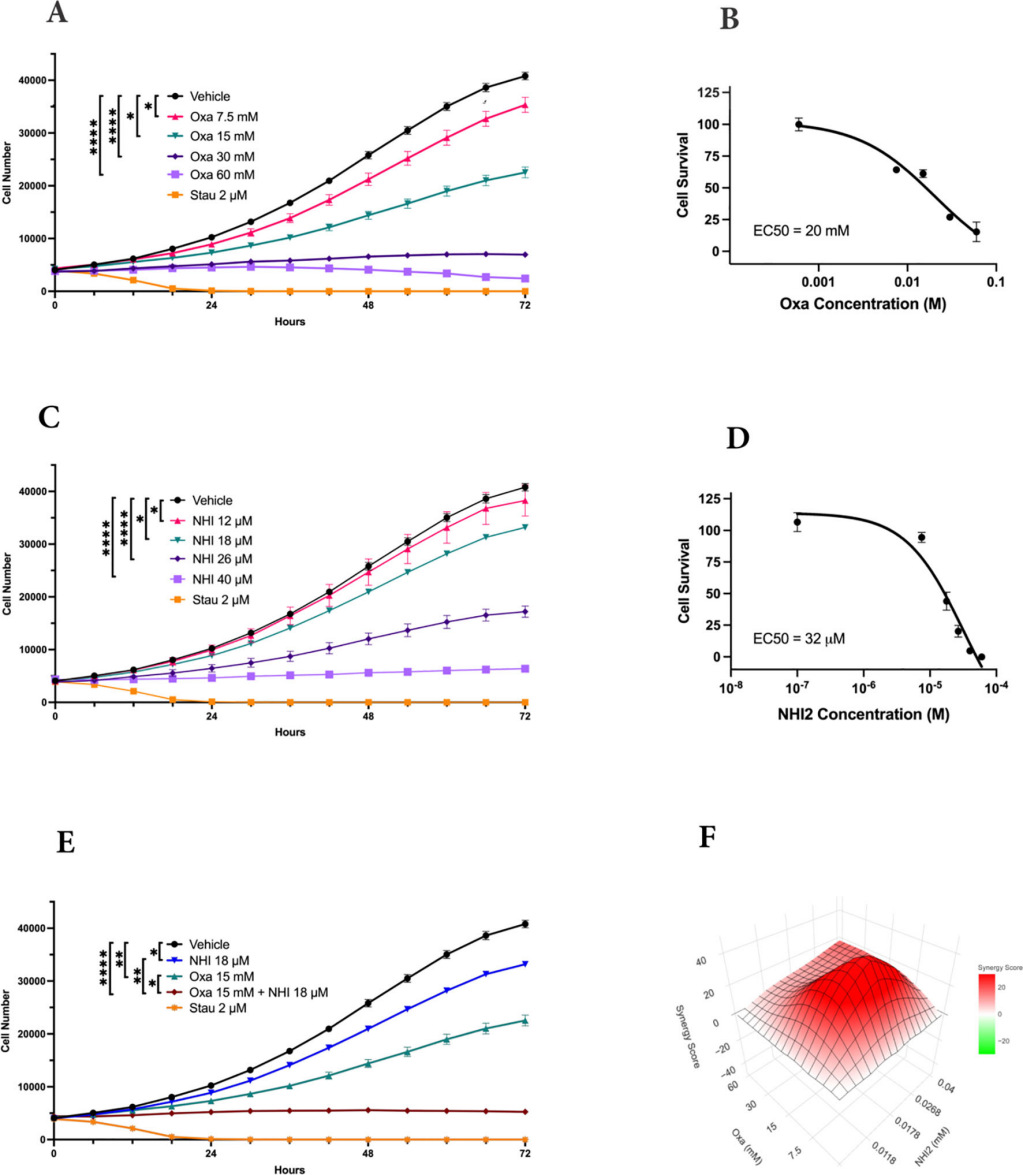
LDH-inhibitors oxamate and NHI-2 exhibit potent and synergistic anti- proliferative effects on B78-D14 melanoma cells. Cell proliferation was monitored in real time with the continuous presence of **(A)** Oxa (Oxamate) and **(C)** NHI-2 treatments for 72 hours using an IncuCyte ® S3 instrument. The changes in cell number are used as a surrogate marker of cell proliferation. Data shown are the mean ± SEM (n = 3). Cell viability curves and EC50 values for B78 cell lines treated with **(B)** Oxa and **(D)** NHI-2 for 48 h at various concentrations. EC50 values were calculated in GraphPad Prism using the non-linear fit curve method. **(E)** The graph and statistical analysis demonstrate the enhanced effectiveness of Oxa and NHI-2 combination treatments on B78 cell proliferation compared to single-agent usage, as indicated by the concentrations on the graph (Two-way ANOVA; ****p < 0.0001; **p < 0.01; *p < 0.05). Stau (Staurosporine) was used as positive control. **(F)** Synergy analysis for the Oxa and NHI-2 combination treatment in the B78 cell line, conducted using synergyfinderplus.org. The results are compared using three distinct synergy correlation method: ZIP (zero interaction potency) model, with a synergy score Mean of 20.88 (p < 2e−324). A synergy score exceeding 10 indicates a pronounced synergistic effect.

The impact of LDHIs, specifically Oxa and NHI-2, on the proliferation of B78 (mouse amelanotic melanoma B78-D14), B16-F10 (mouse skin melanoma), and M21 (human metastatic melanoma) cell lines, was screened via real-time cell counting through an IncuCyte™ S3 live cell imaging system. The anti-proliferative effects of Oxa and NHI-2 became evident within 12 hours. Higher concentrations of Oxa (30 and 60 mM) led to a complete blockade of B78 cell proliferation (**Figure 1A**), and EC50 value was calculated as 20 mM for Oxa (**Figure 1B**). NHI-2 was effective in μM levels and 40 μM NHI-2 led to a complete blockade of B78 cell proliferation (**Figure 1C**). The EC50 value was calculated as 25 μM for NHI-2, according to inhibition of B78 cell proliferation (**Figure 1D**). Consistent outcomes were replicated in B16 and M21 cell lines (**Supplementary Figure S1**). At lower doses, such as 15 mM Oxa or 18 μM NHI-2, their combined application demonstrated an effective suppression of B78 cell proliferation, surpassing the outcomes observed with either vehicle control or single-drug treatments (**Figure 1E**).

The synergistic effects of the combination treatment of Oxa and NHI-2 on melanoma cell proliferation were assessed by subjecting cells to various concentrations of these inhibitors, administered either individually or in combination. Normalized results were analyzed using the SynergyFinderPlus tool, incorporating diverse models such as highest single agent (HAS), Bliss independence, Loewe additivity, and zero interaction potency (ZIP) to compute the synergy score (42). The recently developed ZIP model combines the strengths of the Loewe and Bliss models, assuming that non-interacting medicines cause modest modifications in their dose-response curves (43). The ZIP model analysis revealed an average synergy score of 20.88 (*p* < 2e−324) for the interaction between Oxa and NHI-2 in B78 cells (**Figure 1F**). Scores exceeding 10 indicated a synergistic effect. The synergy score reached up to 40 for certain concentrations, such as a combination of 15 mM Oxa and 18 μM NHI-2. Consistent with this, both the HSA and Bliss models affirmed the synergistic impact of the Oxa and NHI-2 combination treatment in B78 cells (**Supplementary Figure S2**). While M21 cells displayed less sensitivity to LDHIs than B78 cells, the combination of Oxa and NHI-2 still exhibited a synergistic effect, with an average ZIP score of 14.17 (*p* = 1.08e−23) (**Supplementary Figure S3**).

Overall, the interaction landscape indicates that the combined treatment of Oxa and NHI-2 exerts a synergistic effect on the survival of melanoma cell lines.

### 2.2 Impact of LDH inhibitors on cell viability and cell cycle in B78 melanoma cells

**Figure 2:**
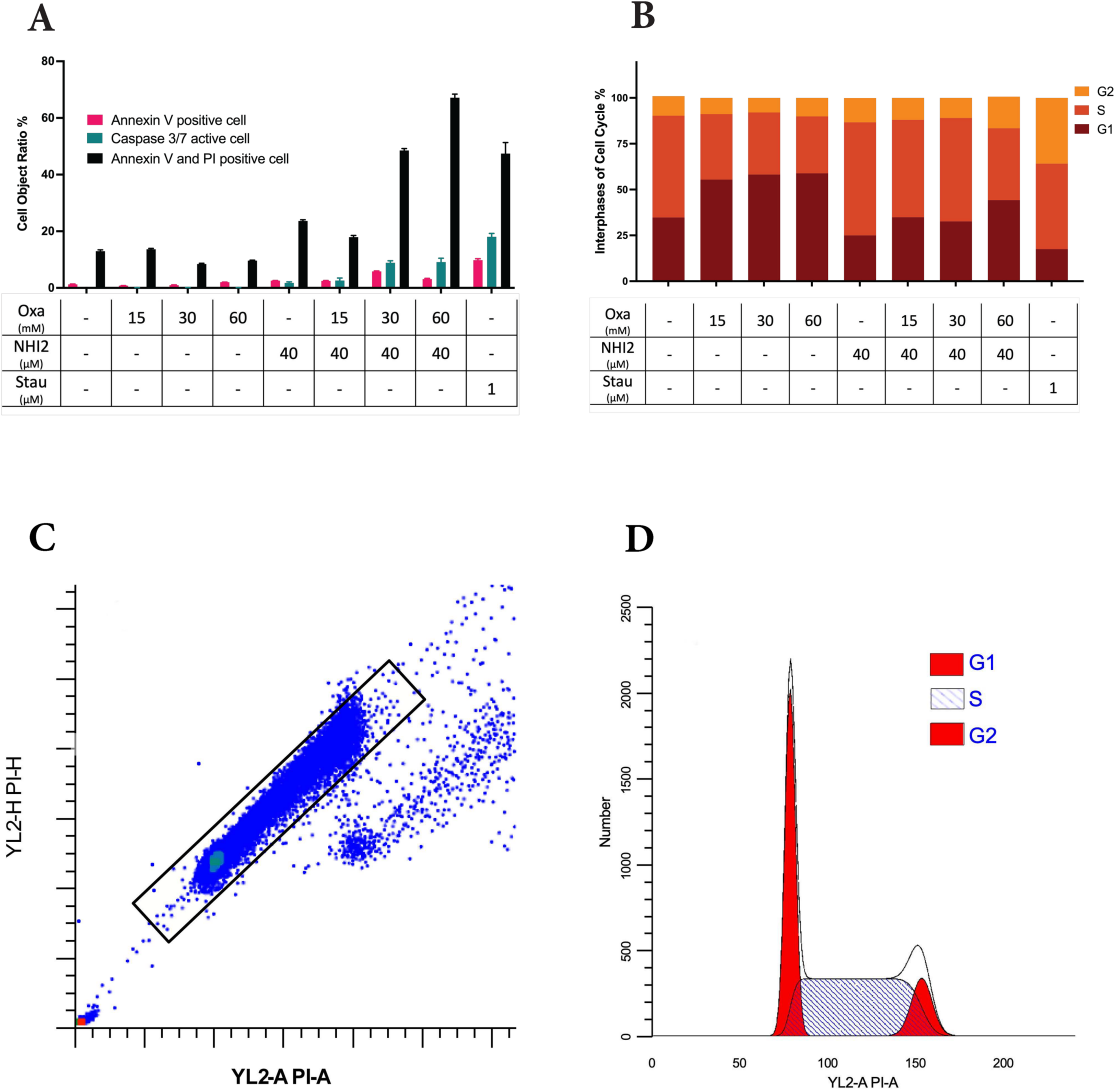
Higher doses of Oxamate and NHI-2 are required to induce apoptosis and disrupt the cell cycle in B78 cells. **(A)** Caspase-3/7 activation was assessed using the IncuCyte^®^ live-cell analysis system, and apoptotic cell percentages were calculated alongside total cell numbers. Annexin V and/or PI-positive cell ratios were determined in non-fixed B78 cells at 24 hours post-treatment via flow cytometry analysis. Data represents the mean of 3 independent wells per condition ± SEM, with a noticeable increase in apoptotic and dead cell ratios observed only at higher doses of Oxa and NHI-2 combined treatment. **(B)** Cell cycle phases were assessed by flow cytometry analysis after PI staining of fixed cells. Data represents the mean of 3 independent repeats per time condition ± SEM. The untreated control sample serves as a reference for **(C)** the gating strategy and **(D)** the quantification model for the G1, S, and G2/M phases of the cell cycle. Analysis was performed using ModFit LT V5.0.9 software. Notably, Oxa treatment led to the accumulation of cells primarily in the G1 phase, whereas NHI-2 treatment resulted in the accumulation of cells in the S phase of the cell cycle.

To assess the impact of LDHIs on apoptosis, apoptotic cell ratios were calculated using NucView^®^ caspase-3 substrate. This substrate allows the detection of caspase-3/7 activity in live cells via the IncuCyte^®^ imaging system. While individual administration of Oxa and NHI-2 did not induce a notable rise in caspase-3/7 activation, a significant increase in apoptosis was observed when 40 μM NHI-2 was combined with either 30 or 60 mM Oxa in B78 cells (**Figure 2A**). Specifically, 24 hours after treatment, the apoptotic cell ratio increased to 8.88% in the 30 mM Oxa + 40 μM NHI-2 group and to 9.12% in the 60 mM Oxa + 40 μM NHI-2 group, compared with 0.14% in the vehicle control group. Lower doses of the drugs, even in combination, did not significantly alter the apoptotic cell ratio, according to caspase-3/7 activity (**Supplementary Figure S4**).

To further investigate apoptosis and cell death, cells were treated with various concentrations of Oxa and NHI-2 for 24 hours. Subsequently, Annexin V/PI staining and flow cytometric analysis were conducted. The flow data, consistent with caspase-3/7 results, demonstrated an increase in early apoptotic (Annexin V positive) cell ratios, particularly with the combination of 40 μM NHI-2 with either 30 or 60 mM Oxa (**Figure 2B**). The early apoptotic cell ratio was 1.25% for the vehicle treatment, 5.82% for 30 mM Oxa + 40 μM NHI-2, and 3.09% for 60 mM Oxa + 40 μM NHI-2. Furthermore, the late apoptotic/dead (Annexin V + PI positive) cell ratio moderately increased in the 40 μM NHI-2 group to 23.64%, and it significantly increased in the 30 mM Oxa + 40 μM NHI-2 to 48.53% and 60 mM Oxa + 40 μM NHI-2 to 67.15% groups compared to the vehicle control, which was at 12.98%.

Furthermore, alterations in cell cycle distribution in B78 cells were analyzed after 24 hours of treatment with Oxa and NHI-2 either alone or in combination. Flow cytometry was employed to analyze changes in the cell cycle after PI staining. The results demonstrated that Oxa induced accumulation in the G1 phase of the cell cycle, whereas NHI-2 led to an increased cell population in the S and G2 phases, as evidenced by the gating strategy and quantification model employed (**Figures 2C, D**).

Together, these findings suggested that Oxa and NHI-2 may affect distinct pathways, and their combination has detrimental effects on B78 cells.

### 2.3 Metabolic impacts of Oxa and NHI-2 on glycolytic and oxidative pathways

**Figure 3:**
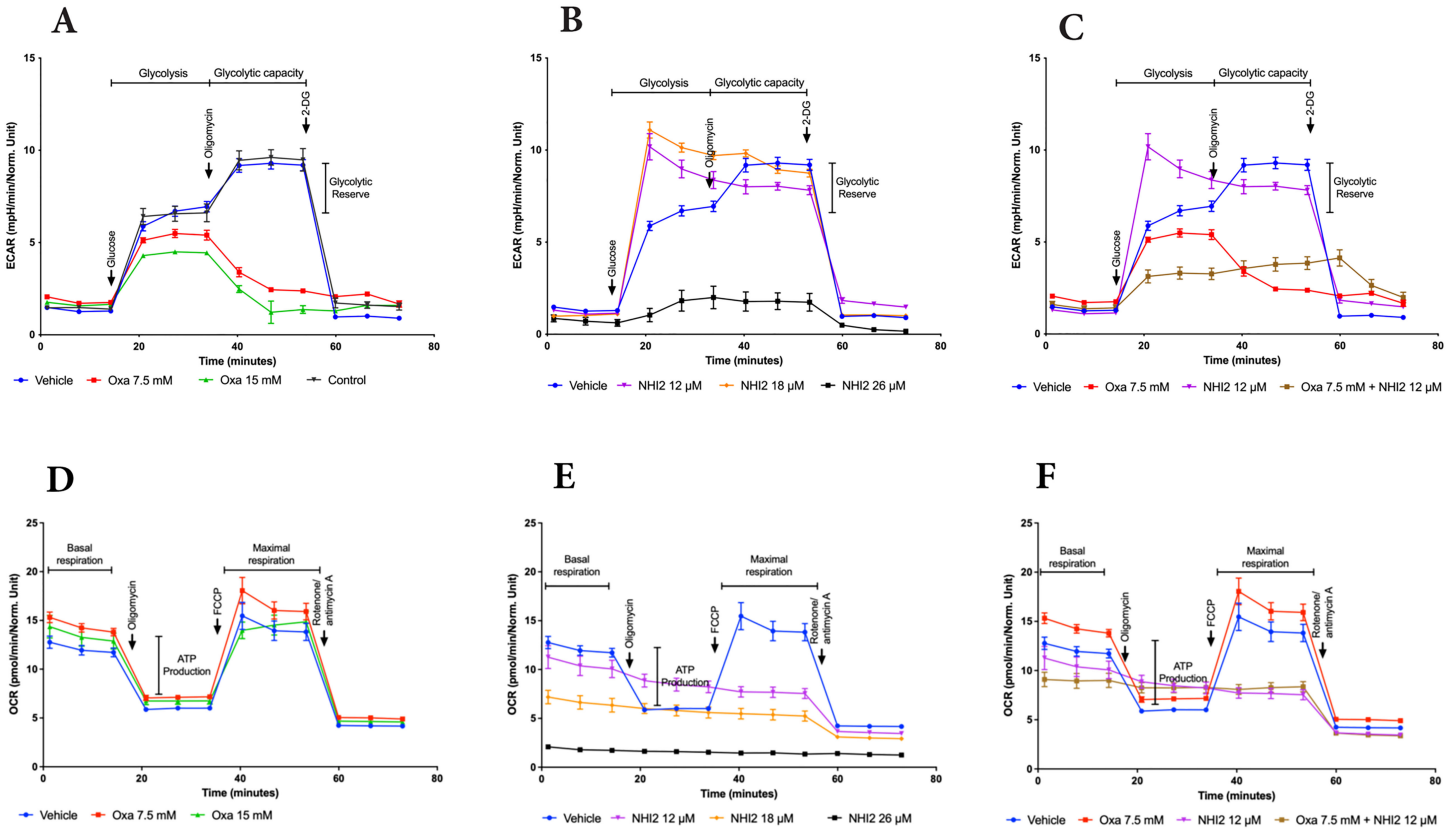
Oxamate and NHI-2 represent distinct effects on cellular bioenergetics. The continuous extracellular acidification rate (ECAR) values were monitored and analyzed employing a Seahorse XFe96 extracellular flux analyzer. B78 cells were treated with Oxa and NHI-2 one hour prior to loading the plate into the analyzer. The arrows indicate the time points of injections by the instrument. Sequential injections of 5 mM glucose, 1 µM oligomycin, and 50 mM 2-deoxy-D-glucose (2-DG) were introduced into the medium. Representative data are presented for the application of **(A)** Oxa, **(B)** NHI-2 as single agents, and **(C)** the combination of both. The continuous oxygen consumption rate (OCR) values were monitored and analyzed employing an XF94 extracellular flux analyzer. B78 cells were treated with Oxa and NHI-2 one hour prior to loading the plate into the analyzer. The arrows indicate the time points of injections by the instrument. Sequential injections of 1 µM oligomycin, and 0.5 μM FCCP (and 0.5 µM Rotenone/Antimycin A were introduced into the medium. Representative data are presented for the application of **(D)** Oxa, **(E)** NHI-2 as single agents, and **(F)** the combination of both. Error bars represent the mean ± standard error of the mean (SEM) (n = 5).

To elucidate the metabolic alterations induced by Oxa and NHI-2 in B78 cells, the extracellular acidification rate (ECAR) and mitochondrial oxygen consumption rate (OCR) were quantified using a Seahorse XFe96 analyzer. Oxa treatment at 7.5- and 15-mM concentrations resulted in a dose-dependent decrease in ECAR, indicating reduced glycolytic activity. Furthermore, cells pre-treated with Oxa displayed a diminished ECAR upsurge in response to the ATP synthase inhibitor oligomycin, suggesting a compromised glycolytic compensatory mechanism (**Figure 3A**). NHI-2 at 12-and 18-μM concentrations elevated the basal glycolytic rate as reflected by ECAR measurements. However, at a 25 μM concentration, NHI-2 significantly decreased both basal and oligomycin-stimulated glycolytic processes (**Figure 3B**). Crucially, co-treatment with Oxa and NHI-2 led to an effective suppression of glycolysis at even lower doses, as evidenced by the ECAR data (**Figure 3C**, **Supplementary Figure S5**).

OCR data demonstrated that Oxa moderately increased both basal and maximal mitochondrial respiration (**Figure 3D**). NHI-2 significantly inhibited basal respiration in a dose-dependent manner, rendering cells unresponsive to FCCP (carbonylcyanide-p-trifluo romethoxy-phenyl-hydrazone) stimulation (**Figure 3E**). The combination of low doses of Oxa (7.5 mM) and NHI-2 (12 μM) resulted in a modest additional reduction in basal respiration compared to NHI-2 alone, likely due to the already substantial efficacy of NHI-2 in inhibiting basal respiration even at low doses as a single agent (**Figure 3F**).

Overall, ECAR measurements indicated the enhanced anti-glycolytic efficacy of the combination of Oxa and NHI-2, particularly at lower doses, surpassing the outcomes of individual treatments. Additionally, OCR data reveals that NHI-2 may also suppress oxidative phosphorylation (OXPHOS), suggesting its broader impact on cellular energy production.

### 2.4 Metabolic profile alterations in B78 cells treated with Oxa and NHI-2

**Figure 4:**
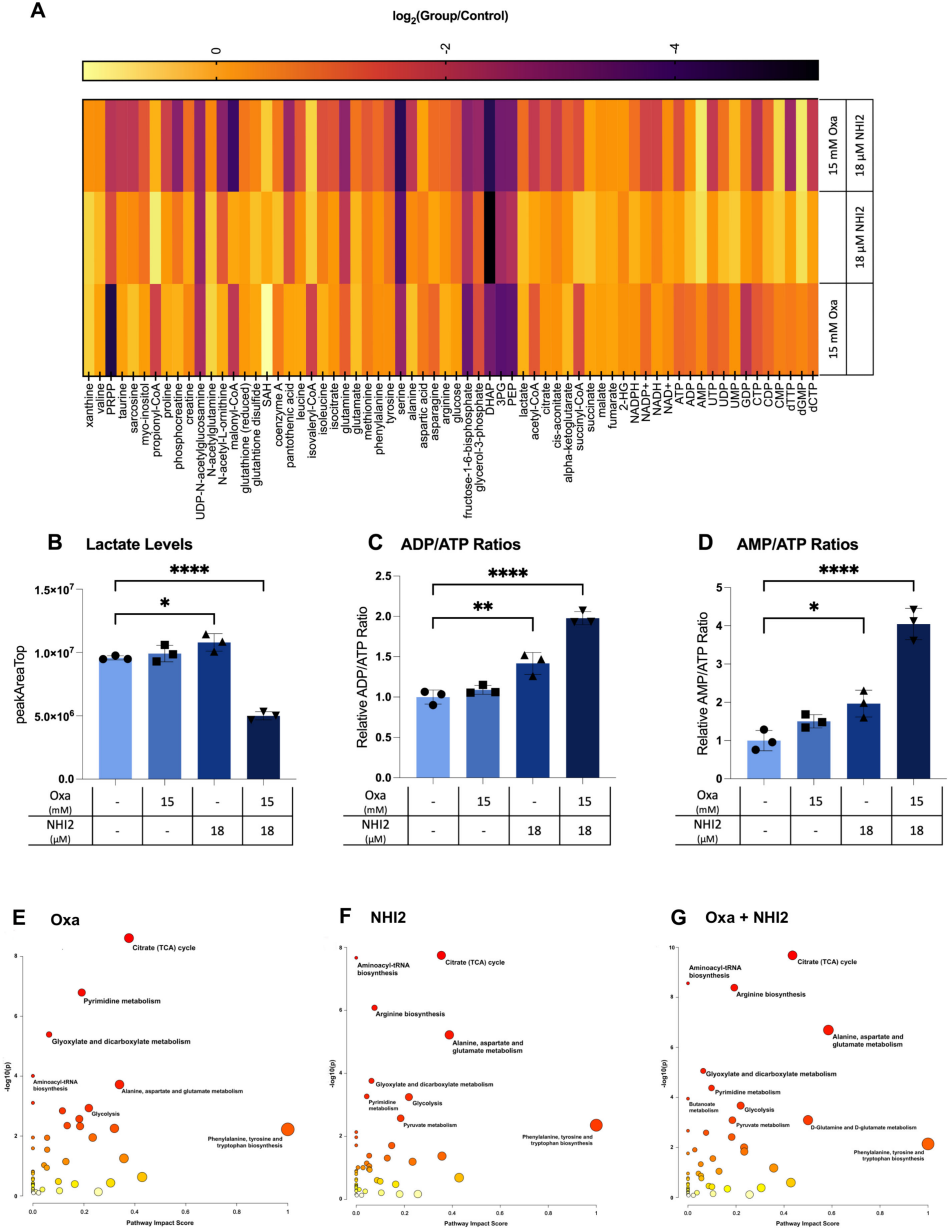
Oxamate and NHI-2 alter metabolite profiles and synergistically impair lactate and ATP production. B78 cells were exposed to drugs 24 hours prior to harvest. Metabolite samples were analyzed using a Thermo Q-Exactive mass spectrometer coupled to a Vanquish Horizon UHPLC. The metabolites reported were identified based on exact m/z and retention times determined with chemical standards. Data were analyzed with MAVEN. To quantify changes in relative metabolite levels, metabolite abundance measured by ion count in LCMS analysis were normalized to total protein content determined by BCA. **(A)** Log2 fold changes between the treatment and control groups represented in heatmap. Intracellular lactate, AMP, ADP, and ATP levels were quantified using LC-MS. Relative levels of **(B)** lactate, ATP, ADP, and AMP in B78 cells were expressed as peak areas normalized to the protein content. **(C)** ADP/ATP and **(D)** AMP/ATP ratios were calculated using the peak area data normalized to the protein content. Values represent the mean +/- SD of three technical runs for each sample. Statistical analysis was performed using one-way ANOVA; titititi *p* < 0.0001; titi*p* < 0.01; ti*p* < 0.05. The web-based platorm, MetaboAnalyst 5.0, was employed for pathway analysis, with enriched pathways predicted through the mummichog algorithm. Graphs illustrate the impacted pathways for **(E)** 15 mM Oxa, **(F)** 18 μM NHI-2, and **(G)** combination treatment groups. The sizes of the data points are based on their x values, while the colors correspond to their y values.

To understand the effects of Oxa and NHI-2 treatments on metabolic pathways, metabolite levels in B78 cells were analyzed using liquid chromatography–mass spectrometry (LC-MS). This approach enabled the detection and quantification of sixty-six intracellular metabolites (**Figure 4A**). Only the combination treatment of Oxa (15 mM) and NHI-2 (18 μM) resulted in a significant reduction in intracellular lactate levels, underscoring the importance of inhibiting LDHA and LDHB enzymes concurrently for efficient suppression of lactate production (**Figure 4B**). Increased ratios of ADP/ATP and AMP/ATP, especially evident in the combination treatment group when compared to the untreated control, suggest cellular energy depletion (**Figures 4C, D**). This state of energy depletion could account for the observed significant inhibition of cell proliferation. The KEGG pathway analysis of altered metabolites revealed that Oxa and NHI-2 had an impact not just on glycolysis but also on other related metabolic processes such as the tricarboxylic acid (TCA)cycle, arginine biosynthesis, pyrimidine metabolism, aminoacyl-tRNA biosynthesis, and alanine, aspartate, and glutamate metabolism (**Figures 4E-G**). The combination treatment of Oxa and NHI-2 exhibited greater significance scores for numerous pathways, as highlighted in **Supplementary Table S1**. The extensive inhibition of metabolic pathways, spanning from energy generation to macromolecule production, likely underlies the robust impact of the Oxa and NHI-2 combination on B78 cells.

### 2.5 Cytokine and chemokine profile modulation following LDH inhibitors treatment

**Figure 5:**
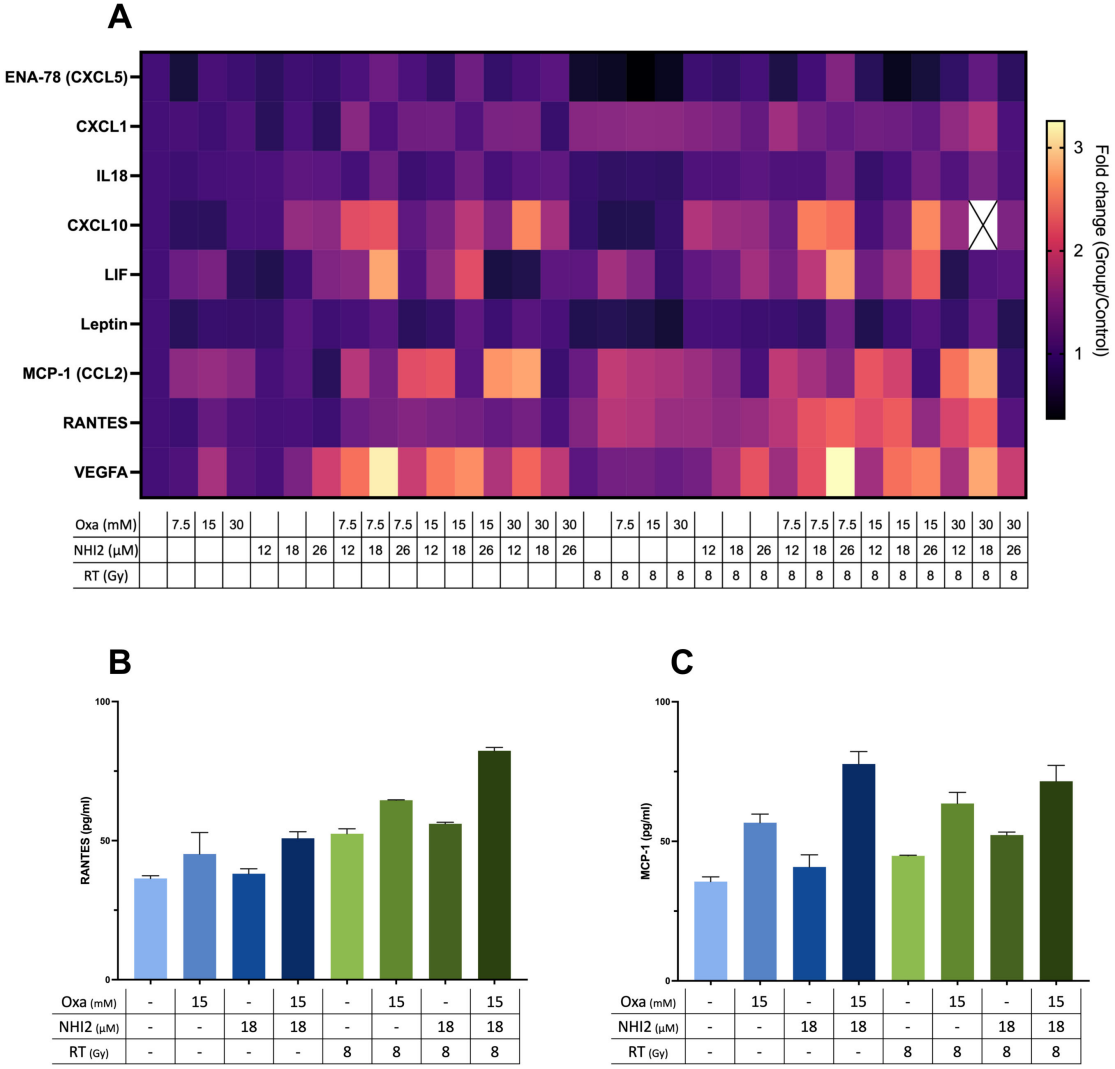
Combined Oxamate and NHI-2 treatment with ionizing radiation upregulates RANTES and MCP-1 expression in B78 cells. Multiplex analysis was conducted using the ProcartaPlex Mouse Immune Monitoring Panel 48plex, allowing for the simultaneous assessment of 48 cytokines, chemokines, and growth factors in B78 cell media following a 24-hour incubation with Oxamate (Oxa), NHI-2, and/or radiation therapy (RT). The data presented show the fold changes in detectable targets relative to the vehicle control. **(A)** Heatmap illustrating alterations in cytokine and chemokine levels in B78 cell media after 24-hour incubation with Oxa, NHI-2, and/or RT. Representative data highlight the levels of **(B)** RANTES and **(C)** MCP-1 in response to different treatments, including Oxa, NHI-2, and RT. Error bars represent the mean ± SEM (n = 2).

Considering the data that highlight the potent anti-proliferative effects of LDHIs but also emphasizing the need for high concentrations to induce cell death, the study explored the potential of using LDHIs in combination to enhance the efficacy of current melanoma treatment regimens. In line with this approach, screening of cytokines, chemokines, and growth factors was conducted to assess potential alterations with LDHI treatments, employing Luminex xMAP technology. The results indicated the modulation of a group of components under LDHI treatments (**Figure 5A**). Particularly noteworthy was the substantial increase observed in RANTES and MCP-1 (**Figures 5B, C**). The elevation of these chemokines holds significant promise due to their positive effects on the recruitment of various immune cells (44, 45). The concurrent increase in these chemokines induced by LDHIs suggests the potential for enhancing immune cell response, making LDHIs promising candidates for further investigation to elevate the efficacy of current immune therapies in the treatment of melanoma.

### 2.6 Enhanced efficacy of immune checkpoint inhibitors via LDH inhibitors

**Figure 6:**
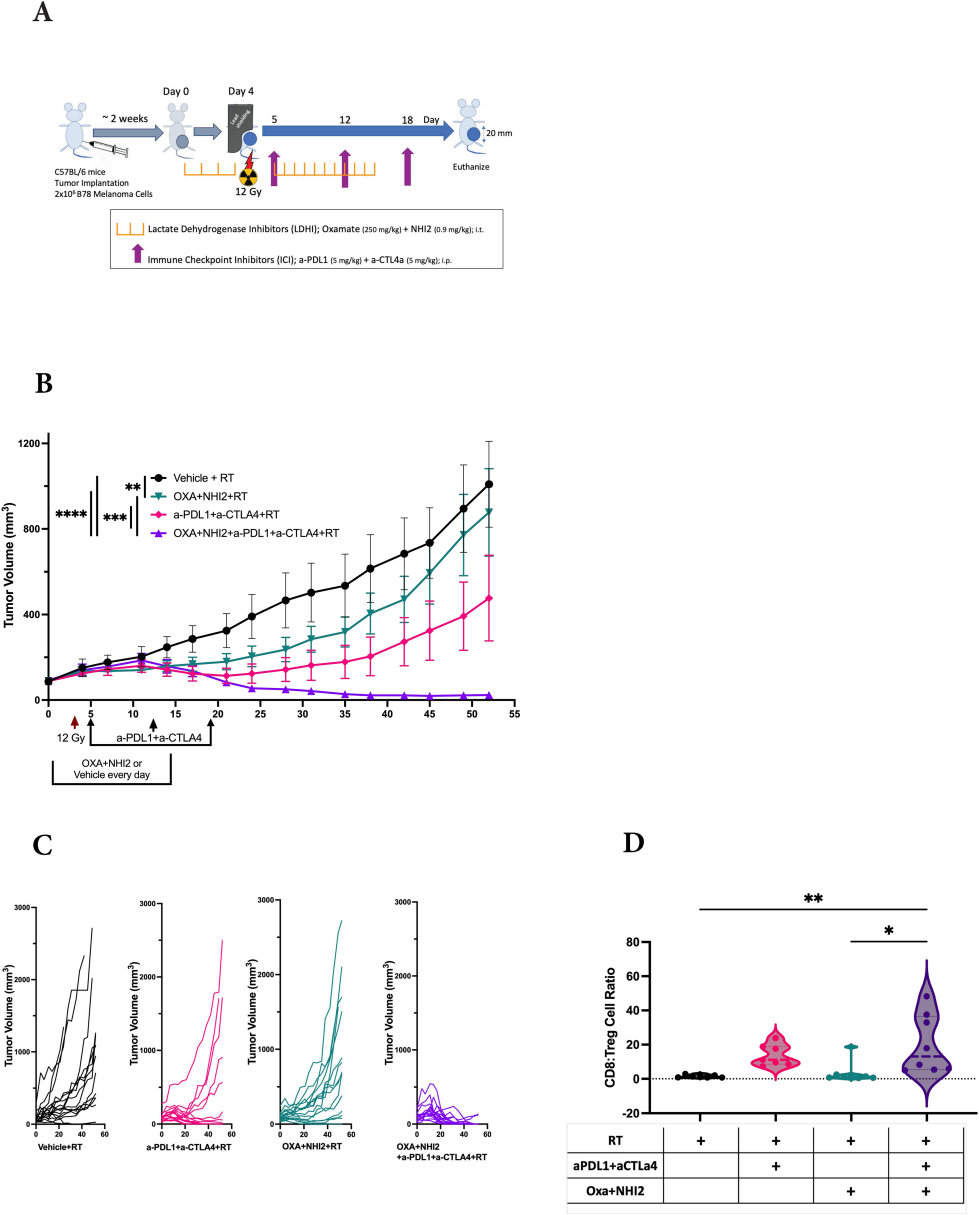
LDH inhibitors increase the therapeutic efficacy of immune checkpoint inhibitors in B78 tumors. **(A)** Schematic representation of the single-tumor mouse model and the treatment protocol. C57BL/6 mice were intradermally implanted with B78 syngeneic melanoma cells. When tumors reached an average volume of ~80 mm3, mice were randomized for intra-tumoral injections of Oxa and NHI-2 (depicted in purple and pink) or PBS (depicted in black and green) daily from day 0 to 15. On day 4, all mice received a 12Gy radiation therapy (RT) applied to the right flank. Anti-PDL1 and anti- CTLA4 (depicted in green and pink) or PBS (depicted in black and purple) were injected intraperitoneally on days 5, 12, and 18. **(B)** Average +/- SEM tumor volume from two individual experiments, total n=16 mice/group. Statistical analysis was performed using one-way ANOVA tests with Tukey *Post-Hoc* test; ****p < 0.0001; ***p < 0.001; **p < 0.01. **(C)** Individual tumor volumes from two individual experiments. **(D)** While no significant differences were observed in the CD8:Treg cell count ratio following RT+ICIs (pink) compared to RT alone (black) or following RT+LDHIs (green) compared to RT alone, the combination of RT+LDHIs+ICIs (purple) led to a significantly increased CD8:Treg cell ratio compared to RT+LDHIs (tip < 0.05) and to RT alone (titip < 0.01). Flow cytometry data represent the compilation of n=8 mice/group. One-way ANOVA tests with Tukey *Post-Hoc* test.

Because we observed increased levels of specific chemokines following Oxa and NHI-2 treatment, the potential of LDHIs to enhance the efficacy of current melanoma treatment regimens, namely ICIs, was tested. The LDHIs+ICIs regimen was assessed using an established B78 melanoma tumor model (**Figure 6A**), where the combination of ICIs and radiation therapy (RT) has previously demonstrated a reduction in tumor development without achieving complete eradication (46). In this study, we administered ICIs at intervals of 6 to 7 days. This dosing schedule was intentionally selected based on recent evidence suggesting that extending the intervals between ICI treatments can reduce adverse effects while maintaining their therapeutic effectiveness (47–51). Additionally, the timing of ICI administration was evaluated using current murine model. The results suggest that a 7-day interval between doses is as effective, if not more effective, than a more frequent 3-day schedule (data not shown). These findings support the feasibility of extending dosing intervals without compromising therapeutic efficacy.

The combined analysis of data from two distinct experiments yielded a total sample size of n=16 per group. In comparison to the RT alone group, the treatment of either LDHIs or ICIs alongside RT individually showed a modest inhibition of tumor growth. However, the treatments combining RT with LDHIs, and RT with LDHIs and ICIs (RT+LDHIs+ICIs) exhibited significantly greater efficacy in reducing tumor growth (**Figure 6B**) and led to the cure of 13 out of 16 mice compared to RT+ICIs (7/16, *p* < 0.05) or RT+LDHIs (2/16, *p* < 0.0001) (**Figure 6C**).

Furthermore, in the evaluation of the immune landscape on day 8 after treatment, only minor changes in the myeloid populations were observed following RT+LDHIs or RT+LDHIs+ICIs treatments (**Supplementary Figure S6**). Similarly, no significant alterations were detected in NK and NKT cell populations with any treatment groups compared to RT alone (**Supplementary Figure S7**). However, a significant enhancement of the ratio of CD8 to T regulatory cells (CD8:Tregs) was detected in the tumor samples of the combination of RT+LDHI+ICIs group compared to RT alone (**Figure 6D**). No significant differences were observed in any other treatment groups compared to RT alone. These data suggest that the addition of LDHIs to RT+ICIs (i.e., RT+LDHIs+ICIs) enhances CD8 effector infiltration—potentially linked to increased RANTES stimulation from B78 melanoma cells—while reducing immunosuppressive Treg cells in the TME. Taken together, these *in vivo* studies demonstrated that the combination of ICIs (a-PDL1 and a-CTLA4) with LDHIs (Oxa and NHI-2) enhances the efficacy of ICIs in a melanoma tumor model.

## 3 Discussion

Cancer metabolism has emerged as a significant area of focus in the development of new treatment regimens for various malignancies. Unlike their non-cancerous counterparts, most cancer cells exhibit a remarkable ability to withstand oxidative and nutritional stresses through metabolic reprogramming. This reprogramming enables cancer cells to meet the high energy and biosynthetic requirements for tumor formation while also playing a role in the development of resistance to various treatments in tumors (17, 52, 53).

This study aimed to target the LDH enzyme, considering the preference of cancer cells to utilize glycolysis for energy production even in the presence of oxygen (Warburg effect) (1) and their ability to adapt their metabolism to changing TME conditions. The results indicated that the LDHA inhibitor Oxa alone could reduce the rate of ECAR but not completely halt it (**Figure 3A**), so it could not block lactate production completely in B78 cells (**Figure 4A**). This aligns with recent findings suggesting that both LDHA and LDHB contribute to pyruvate-to-lactate conversion, requiring the simultaneous knockout of both LDHA and LDHB to stop lactate production in LS174T (human colorectal adenocarcinoma) and B16-F10 (mouse melanoma) cell lines (54). Indeed, NHI-2, an LDHA and LDHB inhibitor, demonstrated significant inhibition of ECAR (**Figure 3B**) and ATP production (**Figures 4C, D**) when applied at a dose of 26 μM in B78 cells. However, NHI-2 was not effective at reducing ECAR levels at lower concentrations (12 and 18 μM). Similarly, NHI-2 exhibited no effect on B78 cell proliferation at lower concentrations. However, a marked decrease in cell proliferation was observed at concentrations beginning from 26 μM. The observed threshold effect, coupled with the low solubility of NHI-2, poses challenges in achieving its effective concentration within tumors. To address this, our study explored the combined application of Oxa and NHI-2, revealing synergistic interactions between the two compounds. This combination effectively inhibited cell proliferation and the production of glycolysis, lactate, and ATP, even at lower concentrations of each compound that were not effective when each compound was applied individually.

Cell cycle analysis revealed that Oxamate induced arrest in the G1 phase, while NHI-2 caused cell accumulation in the S and G2 phases (**Figure 2B**). The energy shortage and altered metabolic environment caused by Oxamate may lead to cell cycle arrest in the G1 phase as the cells are unable to accumulate the necessary energy and biosynthetic precursors required for DNA replication. However, NHI-2, as an LDH inhibitor and NADH competitor, not only affects glycolysis but also impacts mitochondrial metabolism and the NAD+/NADH balance, which are critical during DNA synthesis and cell division. The disruption of these processes during the S phase (DNA replication) and G2 phase (preparation for mitosis) can lead to cell cycle arrest at these points. These results indicated that using Oxa and NHI-2 together could be a promising therapeutic strategy because they exert complementary effects on the cell cycle and metabolism. Oxamate impedes the initial commitment to DNA synthesis, while NHI-2 disrupts later stages of cell division, leading to a comprehensive blockade of cancer cell proliferation. This dual approach could potentiate the effects of each inhibitor, making it more difficult for tumor cells to escape therapy.

Furthermore, metabolic flux analysis and metabolomic results demonstrated that NHI-2 not only inhibited ECAR but also had an impact on OXPHOS. This may be explained by a close link between glycolysis and the TCA cycle, and disruptions in one pathway often have downward effects on the other. Inhibition of LDH can lead to a bottleneck in glycolysis, reducing the supply of substrates (e.g., acetyl-CoA) and cofactors (NAD+) necessary for optimal TCA cycle function. Additionally, NHI-2 acts as a NADH competitor, directly impacting the NAD+/NADH cycle, which may further disrupt enzyme activities within the TCA cycle. Given the ability of cancer cells to alter their metabolic pathways and the cellular heterogeneity within tumors, this dual effect of NHI-2 can be advantageous for therapeutic purposes. Metabolite profiling further confirmed the enhanced effects of the combination treatment on lactate metabolism and revealed broader impacts on other metabolic pathways, providing insights into the mechanisms underlying the anti-proliferative effects of Oxa and NHI-2.

Besides disturbing the energy flow in cancer cells, LDHIs also possess the potential to regulate the immune response within the TME. Recent studies have highlighted that lowering lactate levels in the tumor can enhance immune responses, primarily by boosting the activity of immune cells through the reduction of acidity in the TME (21, 28, 55). Studies have also shown that metabolically active tumor cells can outcompete T cells for essential nutrients such as glucose and glutamine, leading to T cell exhaustion and reduced efficacy of immune responses (56, 57). NHI-2, through its dual effects, may disrupt the metabolic activity of tumor cells, thereby reducing this metabolic competition and enhancing T cell-mediated immune responses. Furthermore, a recent study has introduced a novel perspective, suggesting that LDHIs may directly regulate the immune response by inhibiting suppressive immune subsets (23). Given the observed outcomes, this study explored the potential of targeting LDH to improve the TME for ICI treatments in melanoma. The B78 melanoma tumor model was employed to evaluate the combination treatment of LDHIs and ICIs, revealing a significant enhancement in treatment efficacy upon the incorporation of LDHIs into the ICIs treatment regimen.

While ICIs have revolutionized cancer treatment, the resistance to these therapies remains a significant challenge. Therefore, novel approaches to enhance ICI efficacy are needed. The findings of this study have important implications for the development of new therapeutic strategies for melanoma. Targeting LDH could enhance the efficacy of ICIs and RT, potentially leading to more robust and durable patient responses. By understanding the complex interplay between lactate metabolism and immune cell modulation, new approaches can be explored to overcome the limitations of current treatments and improve patient outcomes.

The development and utilization of more specific LDHIs will further validate the specificity and safety of the effects observed in this study, opening the way for clinical trials. Such trials could assess the efficacy of combining LDHIs with ICIs and RT in a clinical setting, potentially leading to the approval of new treatment protocols. Moreover, these findings provide valuable insights into novel therapeutic strategies for cancer management, not only for melanoma but potentially for other cancer types that exhibit similar metabolic characteristics.

The integration of anti-metabolic approaches with immunotherapy holds great promise for advancing cancer treatment. This combination strategy could be particularly beneficial in overcoming resistance mechanisms that limit the effectiveness of current therapies. As such, our study lays the foundation for future research exploring the therapeutic potential of targeting lactate metabolism, which could ultimately lead to improved clinical outcomes for a broader range of cancer patients.

## 4 Materials and methods

### 4.1 Study design

The study was designed to evaluate how LDHIs both directly hinder melanoma cell growth and enhance anti-tumor immunity when combined with ICIs in a preclinical model. This objective was addressed by (i) detecting the impact of Oxamate and NHI-2, individually or combined, on melanoma cell proliferation, viability, and cycle by further exploring changes in metabolic pathways, metabolites, and cytokine profiles in B78 melanoma *in vitro*, and (ii) assessing the efficacy of the combination of LDHIs and ICIs on a B78 melanoma tumor model.

Sample sizes were determined with the Biostatistics and Bioinformatics Core at University of Wisconsin-Madison to ensure that all studies were sufficiently powered, and that data were properly analyzed and interpreted. The exact sample size (n numbers) and statistic methods used in each experiment are indicated in the respective figure legends. The drug concentrations presented in the manuscript were determined by the authors based on preliminary cell-based experiments and initial findings from a pilot animal study. Cell-based assays were conducted in triplicate to ensure reproducibility, whereas animal studies were replicated twice, with the resulting data being aggregated for analysis. In the animal experiments, mice were randomized according to tumor volume at the initiation of the treatment protocol (about 80 mm^3^). Body weights were measured on a weekly basis. Tumor dimensions and overall health assessments were performed twice weekly, and mice were euthanized when any mean tumor diameter reached or exceeded 20 mm. The study tasks — including randomization, administration of treatments, tumor sizing, and health evaluations — were conducted by different individuals under a double-blind arrangement. All animal experiments were in accordance with the guidelines of the Institutional Animal Care and Use Committee at the University of Wisconsin-Madison. Immune profiling experiments were performed blinded by a person without the knowledge of the treatment conditions.

### 4.2 Cell lines

B16-F10 [Mouse skin melanoma], M21 [Human metastatic melanoma] and the B78-D14 cell line were obtained from Dr. Paul Sondel (University of Wisconsin-Madison). Cells were grown in RPMI 1640 or DMEM (Gibco) media and supplemented with 10% fetal bovine serum (FBS), 2 mM L- glutamine, 100 U/mL penicillin, and 100 μg/mL streptomycin. Cells were maintained in culture below 80% confluence for all passages, and early passages after thaw (3–8) were used for all experiments. Cell authentication was performed per American Type Culture Collection guidelines using morphology, growth curves, and mycoplasma testing within 6 months of use.

### 4.3 IncuCyte imaging for dynamic cell proliferation and apoptosis analysis

To facilitate tracking of cell proliferation, a lentivirus-based method was employed to introduce a nuclear-localized fluorescent protein/mKate2, into the target cells, ensuring bright and consistent labeling throughout (**Supplementary Figure S4**). Subsequently, cells were cloned to obtain a population with uniformly labeled nuclei. Proliferation assays were performed using the IncuCyte live cell imaging system (Sartorius, Germany). This system enables real-time cell counting by analyzing florescence-labelled cell images over time. Briefly, 2×10^3^ cells were seeded in 96-well TC-treated microplates in 100 μL culture medium containing 5 mM glucose and grown for 24 h. The next day, the cells were exposed to LDH inhibitors and subsequently placed in the IncuCyte live cell imaging system. Simultaneously, NucView^®^488 Caspase-3 Enzyme Substrate (Biotium, Fremont, CA) were added to wells to determine the rate of apoptotic cells. Entire well images were captured at 6-hour intervals over the course of 4 consecutive days. This continuous imaging provided a dynamic view of total and apoptotic cell numbers over time. Each experiment consisted of three independent replicates. The cell numbers were plotted and analyzed by GraphPad Prism software.

### 4.4 Flow cytometric detection of apoptosis and cell death

FITC Annexin V Apoptosis Detection Kit I (BD Biosciences, San Jose, CA) was used to detect apoptosis by flow cytometry. Briefly, cells were trypsinized and washed twice with cold PBS, and then resuspended in 1X Binding Buffer at a concentration of 1 x 10^6^ cells/mL. Next, 100 µl of this cell suspensions (equivalent to 1 x 10^5^ cells) were transferred to a 5-mL culture tube, and 5 µl each of FITC Annexin V and PI were added and then were incubated for 15 min at room temperature in the dark. 400 µl of 1X Binding Buffer was added, and the cells were promptly analyzed by flow cytometry using an Attune NxT flow cytometer and analyzed using FlowJo version 10.7.1. Annexin V is a calcium-dependent phospholipid-binding protein that has a high affinity for the membrane phospholipid phosphatidylserine (PS) and is useful for identifying apoptotic cells with exposed PS. Propidium Iodide (PI) is a standard flow cytometric viability probe and is used to distinguish viable from nonviable cells. Viable cells with intact membranes exclude PI, whereas the membranes of dead and damaged cells are permeable to PI. Cells that stain positive for FITC Annexin V and negative for PI are undergoing apoptosis. Cells that stain positive for both FITC Annexin V and PI are either in the end stage of apoptosis, are undergoing necrosis, or are already dead. Cells that stain negative for both FITC Annexin V and PI are alive and not undergoing measurable apoptosis.

### 4.5 Flow cytometric detection of cell cycle

Cells were harvested and then resuspended in PBS. Cold ethanol was added dropwise to the pelleted cells to achieve a final concentration of 70% and fixed on ice for two hours. Cells were washed in PBS and were resuspended in staining buffer [0.1% Triton X-100, RNase A (100 μg/mL) and PI (50 μg/mL)]. Samples were incubated overnight at 4°C in the dark. Data acquisition was carried out on an Attune NxT flow cytometer and analyzed using ModFit 5.

### 4.6 Real-time cell metabolic analysis

Metabolic characterization of B78 cells was performed using a Seahorse XFe96 Extracellular Flux Analyzer (Agilent Technologies, Santa Clara, CA). Mitochondrial stress and glycolytic parameters were measured via Oxygen Consumption Rate (OCR) and ECAR, respectively. Briefly, 2×10^3^ cells were seeded into the Seahorse XF Cell Culture Microplate in 100 μL RPMI-1640 medium containing 5 mM glucose, one day before the experiment. For analysis, cells were resuspended in seahorse XF RPMI medium supplemented with 5 mM glucose (Sigma-Aldrich), 1 mM pyruvate (Sigma-Aldrich), and 2 mM glutamine (Sigma-Aldrich). In this step, LDH inhibitors were added to the designated wells with medium. After incubating the plate in a 37°C non-CO_2_ incubator for 45 min, The Cell Mito Stress Test was performed using 1.5 μM oligomycin, 0.5 μM FCCP, 0.5 μM rotenone, and 0.5 μM antimycin A (RotAA) purchased from Agilent Technologies.

Another plate was prepared as described above, but Seahorse XF Base Medium was used for Glycolysis Stress Test. After incubating the plate in a 37°C non-CO_2_ incubator for 45 min, Glycolysis Stress Test was performed using 5 mM Glucose, 1.0 μM oligomycin, and 50 mM 2-Deoxy-D-glucose purchased from Agilent Technologies. Metabolic parameters were exported and calculated according to the manufacturer’s instructions using the Seahorse Wave desktop software (Agilent Technologies).

### 4.7 Metabolite extraction and liquid chromatography-mass spectrometry analysis

B78 cells were washed three times with Phosphate Buffer Saline (PBS) and intracellular metabolites were extracted with cold 80% (v/v) LC-MS grade methanol. Samples were dried under nitrogen gas and resuspended in LC-MS-grade water. Metabolite samples were analyzed using a Thermo Q-Exactive mass spectrometer coupled to a Vanquish Horizon UHPLC. Analytes were separated on a 100 x 2.1 mm, 1.7 µM Acquity UPLC BEH C18 Column (Waters), with a gradient of solvent A (97:3 H2O: methanol, 10 mM TBA, 9 mM acetate, pH 8.2) and solvent B (100% methanol) at 0.2 mL/min flow rate. The gradient was: 0 min, 5% B; 2.5 min, 5% B; 17 min, 95% B; 21 min, 95% B; 21.5 min, 5%. Data were collected in full-scan negative mode. Setting for the ion source were: 10 aux gas flow rate, 35 sheath gas flow rate, 2 sweep gas flow rate, 3.2kV spray voltage, 320°C capillary temperature, and 300°C heater temperature.

The metabolites reported were identified based on exact m/z and retention times determined with chemical standards. Data were analyzed with MAVEN. To quantify changes in relative metabolite levels, metabolite abundance measured by ion count in LC-MS analysis were normalized to total protein content determined by bicinchoninic acid (BCA) assay. The web-based platform, MetaboAnalyst 5.0, was employed for pathway analysis, with enriched pathways predicted through the mummichog algorithm.

### 4.8 Comprehensive profiling of immune factors in B78 cell culture medium

B78 cell culture media, collected after 24 hours of Oxa ± NHI-2 treatment, underwent cytokine, chemokine, and growth factor measurement using the ProcartaPlex™ Mouse Immune Monitoring Panel 48-Plex (ThermoFisher) protocol. Briefly, cell culture supernatants were prepared by centrifuging samples at 1,400 rpm for 10 min at 4°C. The plate was prepped by adding the capture bead mix and washing it using a hand-held magnetic plate washer. Standards and samples were then added to the wells based on the plate map. The plate was shaken at 600 rpm overnight at 4°C. The next day, a washing step was followed by the addition of the biotinylated detection antibody mix to the plate. After shaking at 600 rpm for 30 min at room temperature and washing off unbound antibodies, Streptavidin-PE (SA-PE) was added, and the plate was shaken at 600 rpm for 30 min at room temperature. Final washes were performed before adding reading buffers to the wells. The plate was run on a xMAP™ instrument and analyzed following the operation manual for the Luminex™ instrument.

### 4.9 Animal study

All mouse procedures were conducted in accordance with the Institutional Animal Care and Use Committee at the University of Wisconsin-Madison. C57BL/6 female mice aged 6 to 8 weeks were purchased from The Jackson Laboratory. B78 flank tumors were engrafted by intradermal flank injection of 2 x 10^6^ cells diluted in 100 μL PBS (58). Tumor volume was assessed twice weekly by a blinded observer, and mice were euthanized when tumors exceeded 20 mm in any direction, or mice were assessed to be in distress by changes to posture, activity, or grooming. Tumor size was determined by precision caliper measurement and tumor volume was approximated using the formula (tumor volume in mm^3^) = [(tumor width in mm)^2^ x (tumor length in mm)]/2. Mice were randomized to treatment groups when tumors reached enrollment size (80 mm^3^), which normally required 2-4 weeks following initial tumor implantation. Approximately 10% of mice failed to develop a suitable tumor, and these mice were excluded from randomization. A course of LDHIs was integrated into an ICIs treatment regimen, as described previously (46, 59). The protocol was further optimized by updating the ICI administration strategy, extending the dosing intervals to 6-7 days, based on recent evidence supporting the efficacy of this approach (47–50). The first day of treatment with LDH inhibitors was defined as “day 1”. Intra-tumoral (IT) injections of LDH inhibitors (given daily on days 1-15) were performed via a single percutaneous needle puncture followed by injection of a 100 µL volume with needle redirection to distribute the injected material in the tumor. Oxa and NHI-2 dosages were 250 mg/kg and 1 mg/kg, respectively. radiation therapy (RT) (12 Gy) was delivered to primary B78 tumors using an Xstrahl Small Animal Radiation Research Platform on day 3. Mice were immobilized using custom lead jigs that exposed the dorsal right flank as previously described. Radiation was delivered in one fraction to a maximum dose of 12 Gy. Anti-CTLA-4 (2.5 mg/kg) and anti-programmed cell death-ligand 1 (anti-PD-L1) (5 mg/kg) were administered in 100 μL PBS by intraperitoneal (IP) injection on days 5, 12, and 18.

### 4.10 Flow cytometry method

Tumors were harvested from euthanized mice on day 15 and minced into 1–2 mm pieces. The small tumor pieces were collected into a 50-mL conical tube containing 5 mL of RPMI 1640 + 10% fetal bovine serum, protein transport inhibitors (ThermoFisher), 100μL of DNAse I (2.5 mg/mL, Sigma-Aldrich) and 100 μL collagenase IV (25 mg/mL, Gibco) and placed in an incubator/shaker at 200 RPM at 37°C for 30 min. After dissociation, the tumors were filtered through a 70 μM cell strainer, washed with 10 mL PBS, and centrifuged at 350 x g for 5 min. Cells were resuspended in 500μL PBS, transferred to flow tubes, and incubated with 0.5 μL GhostRed780 (Tonbo Biosciences) at 4°C for 30 min. Samples were then washed with 2 mL of flow buffer (PBS +2% FBS) and centrifuged at 350 x g for 5 min. TruStain FcX™ PLUS (BioLegend) was added to each sample, and samples were divided into 2 separate flow tubes for analysis of a myeloid antibody panel or a T/NK cell antibody panel. The myeloid master mix antibody markers included: CD45-FITC, CD206-PE, F4/80-PE-Dazzle594, CD3-PE-Cy5, Ly6g-PE-Cy7, MHCII-BV510, Ly6C-BV605, CD11b-BV711, CD11c-APC. The T/NK cell antibody panel included a surface stain of: CD45-FITC, CD4-AF700, CD8-BV605, CD25-BV711, NK1.1-PE-Dazzle594, CD3-PECy5; and an internal stain with: FoxP3-PE Cy7 (ThermoFisher). Cells were stained with surface markers for 30 min at 4°C in the dark and then washed in flow buffer. The myeloid panel data were acquired after wash was performed. For the T/NK cell antibody samples after surface staining the cells were then fixed using eBioscience™ Foxp3/Transcription Factor Staining Buffer Set (ThermoFisher) following the manufacturers protocol. Cells were washed in 1X permeabilization buffer and were then incubated in overnight with FoxP3 antibody in the dark at 4°C. The following day, samples were washed once with permeabilization buffer, centrifuged at 500 x g for 5 min, followed by a wash with flow buffer and centrifugation at 500 x g for 5 min. All data were acquired on an Attune™ NxT flow cytometer (Thermo Fisher) with manufacturer provided acquisition software. Data were analyzed using FlowJo version 10.7.1 (FlowJo LLC, Becton Dickinson & Company (BD) 2006-2020). Gates were determined using Fluorescence Minus One (FMOs).

### 4.11 Statistical analysis

Cell proliferation and tumor volume curves were analyzed by simple linear regression followed by one-way ANOVA with Tukey’s *post-hoc* test for statistical considerations. One-way ANOVA followed by Tukey’s *post-hoc* test for multiple comparisons were performed in flow cytometry studies of immune subsets, and to determine statistical differences for cell count and intracellular lactate levels.

## Data Availability

The raw data supporting the conclusions of this article will be made available by the authors, without undue reservation.
