# Supplementary material for: Metabolic modulation of melanoma enhances the therapeutic potential of immune checkpoint inhibitors
Source: Front Oncol. 2024 Oct 1;14:1428802. doi: 10.3389/fonc.2024.1428802 (PMC11491500; doi:10.3389/fonc.2024.1428802)
Supplement: Supplementary file 1 [file DataSheet1.docx]

**List of Supplementary Materials**

Figures S1 to S7

Table S1, A-C

**SUPPLEMENTARY FIGURES**

**Fig. S1. LDH inhibitors Oxamate and NHI-2 exhibit potent anti-proliferative effects on B16-F10 and UCLA-SO-M21 melanoma cells.** B16-F10 (mouse skin melanoma) cell proliferation was monitored in real time with the continuous presence of (**A)** Oxamate (Oxa) and (**B**) NHI-2 treatments for 48 hours using IncuCyte ® S3 instrument**.** Similarly, UCLA-SO-M21(human metastatic melanoma) cell proliferation was monitored in real time with the continuous presence of (**C**) Oxa and (**D**) NHI-2 treatments for 48h using IncuCyte ® S3 instrument**.** The changes in cell number are used as a surrogate marker of cell proliferation. Data shown are the mean ± standard error of the mean (SEM) (n = 4).

**B**

**
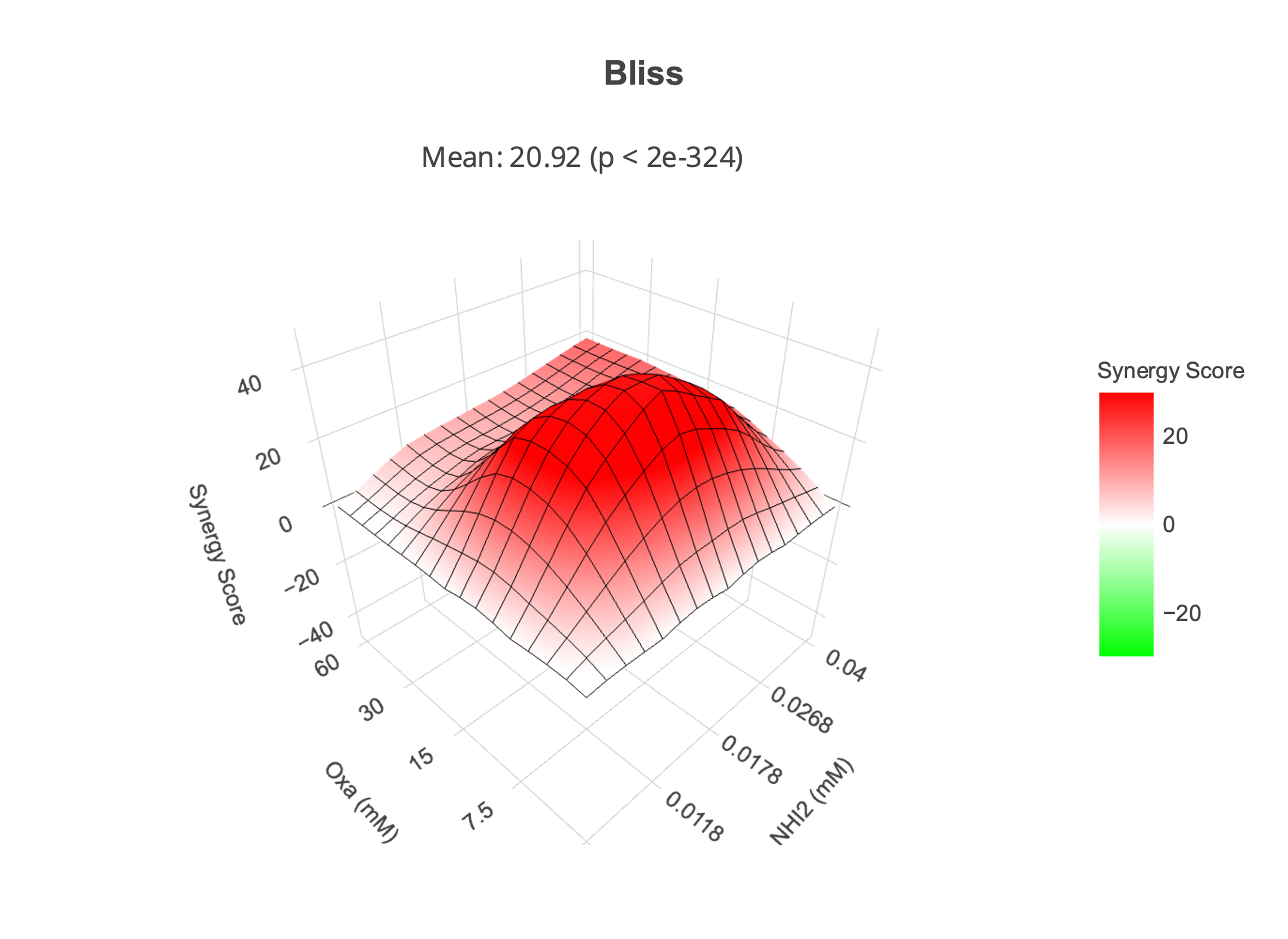
A**

**
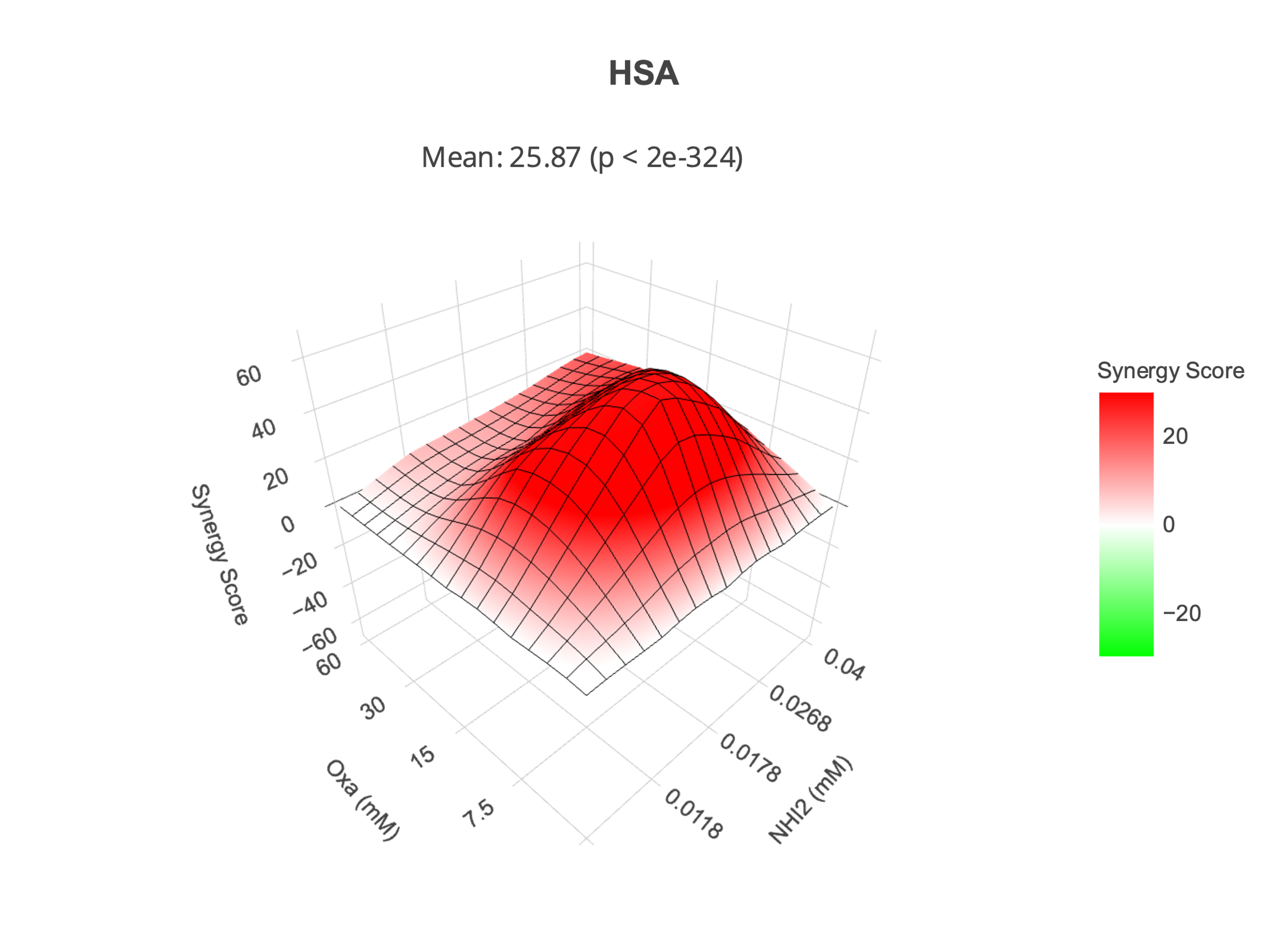
**

**Fig. S2. LDH inhibitors Oxamate and NHI-2 exhibit synergistic anti-proliferative effects on B78-D14 melanoma cells.** Synergy analysis for the Oxa and NHI-2 combination treatment in B78 cell lines, conducted using https://synergyfinderplus.org. The results are compared using three distinct synergy correlation methods: (**A**) HSA model, with a Mean score of 25.87 (*P* < 2e−324); and (**B**) Bliss model, with a Mean score of 20.92 (*P* < 2e−324). A synergy score exceeding 10 indicates a pronounced synergistic effect.


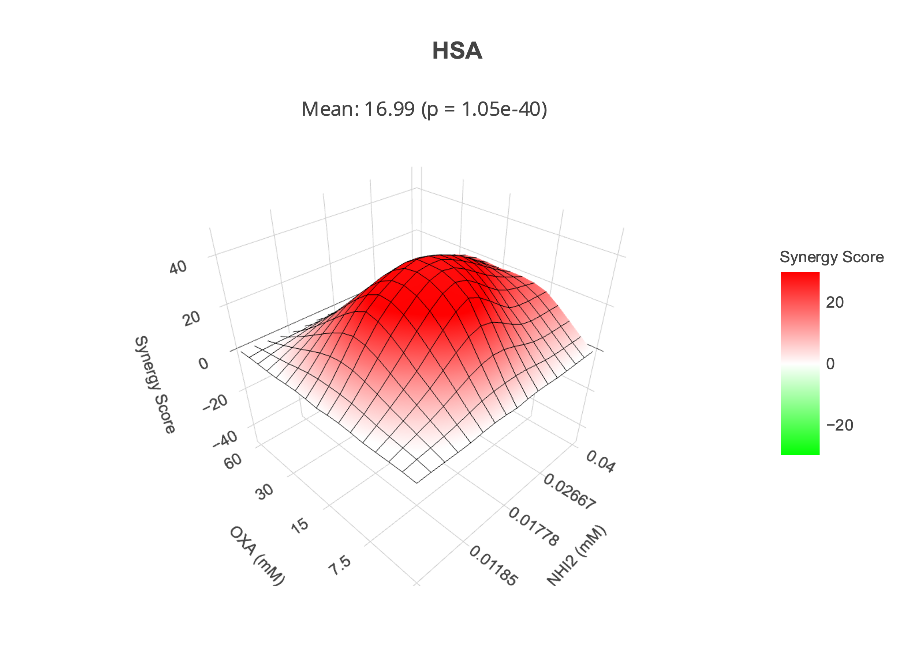


**B**


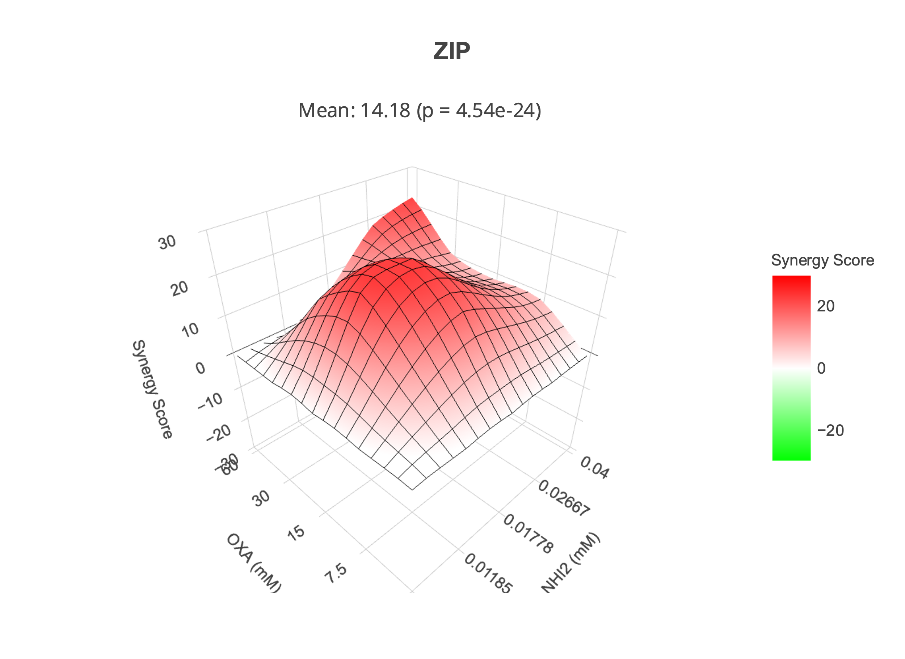


**A**

**C**


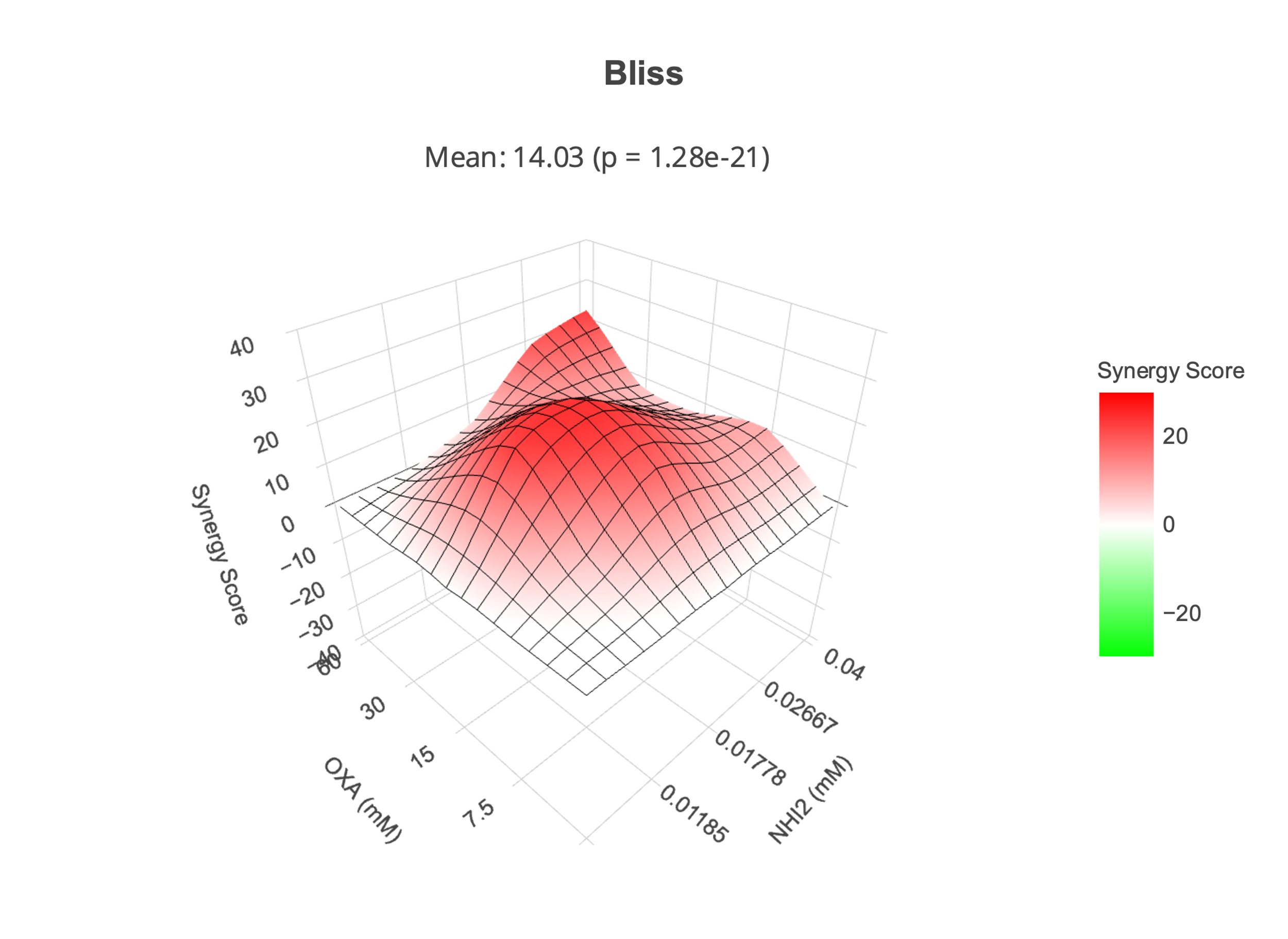


**Fig. S3. LDH inhibitors Oxamate and NHI-2 exhibit synergistic anti-proliferative effects on M21 cells.** Synergy analysis for the Oxa and NHI-2 combination treatment in M21 human melanoma cell lines, conducted using synergyfinderplus.org. The results are compared using three distinct synergy correlation methods: (**A**) ZIP (zero action potency) model, with a synergy Mean score of 14.17 (*P* = 1.08e−23); (**B**) HSA model, with a Mean score of 16.99 (*P* = 1.05e-40); and (**C**) Bliss model, with a Mean score of 14.03 (*P* = 1.28e-21). A synergy score exceeding 10 indicates a pronounced synergistic effect.

**Fig. S4. Lower doses of Oxamate and NHI-2 do not activate apoptosis in B78 cells.** Caspase-3/7 activation was assessed using the IncuCyte® live-cell analysis system, and apoptotic cell percentages were calculated alongside total cell numbers. Data represents the mean of 5 independent wells per condition ± standard error of the mean (SEM), with a noticeable increase in apoptotic cell ratio observed only at positive control (Staurosporine [Stau]) group.

**Fig. S5. Synergistic inhibition of glycolytic parameters by Oxamate and NHI-2 at concentrations ineffective when applied individually.** The continuous extracellular acidification rate (ECAR) values were monitored and analyzed employing an XF94 extracellular flux analyzer. B78 cells were treated with Oxa and NHI-2 one hour prior to loading the plate into the analyzer. The arrows indicate the time points of injections by the instrument. Sequential injections of 5 mM glucose, 1 µM oligomycin, and 50 mM 2-deoxy-D-glucose (2-DG) were introduced into the medium. Representative data are presented for the application of various concentrations of Oxa and NHI-2 as single agents, and the combination of both. Error bars represent the mean ± standard error of the mean (SEM) (n = 5).


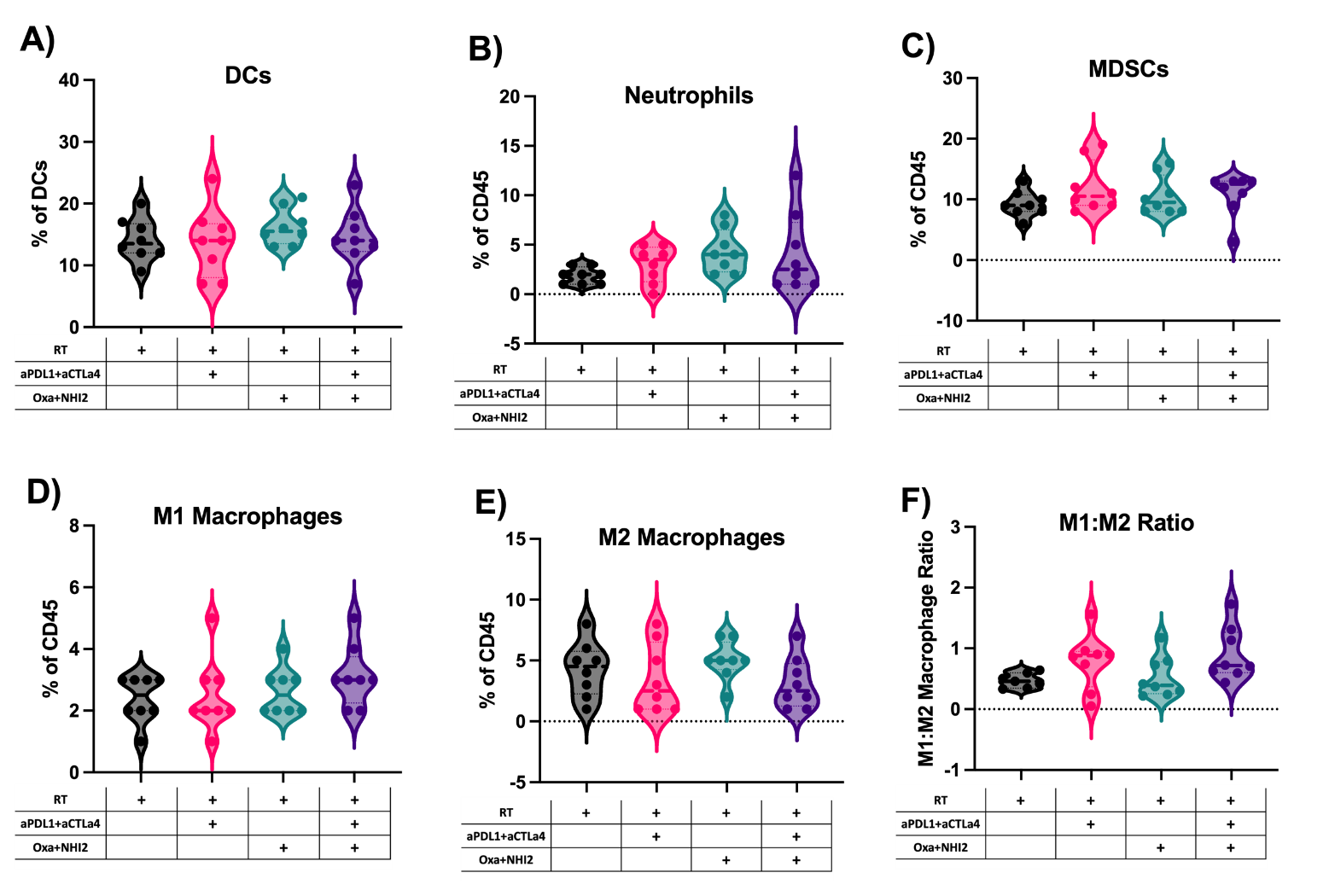


**Fig. S6. Myeloid cell profiling in tumor microenvironment following treatments.** Myeloid populations were assessed for alterations in infiltration into the TME following RT, RT + ICIs, RT + LDHIs or RT + LDHIs + ICIs. There were no significant differences in the infiltration of (**A**) dendritic cells (CD45+/CD3-/CD11b+/CD11c+/MHCII+); (**B**) neutrophils CD45+/CD3-/CD11b+CD11c-/Ly6G+/MHCII-/low/F480-); (**C**) MDSCs (CD45+/CD3-/CD11b+/CD11c-/F480+/low/MHCII-); (**D**) M1 macrophages (CD45+/CD3-/CD11b+/F480+/CD206-/MHCII+); (**E**) M2 macrophages (CD45+/CD3-/CD11b+/F480+/CD206+/MHCII-); or (**F**) in the M1:M2 macrophage cell number ratio based on treatment type. Data represent the compilation of n=4 mice/group, repeated 2 separate times, for a total of 8 mice/group. One-way ANOVA tests with Tukey Post-Hoc test.


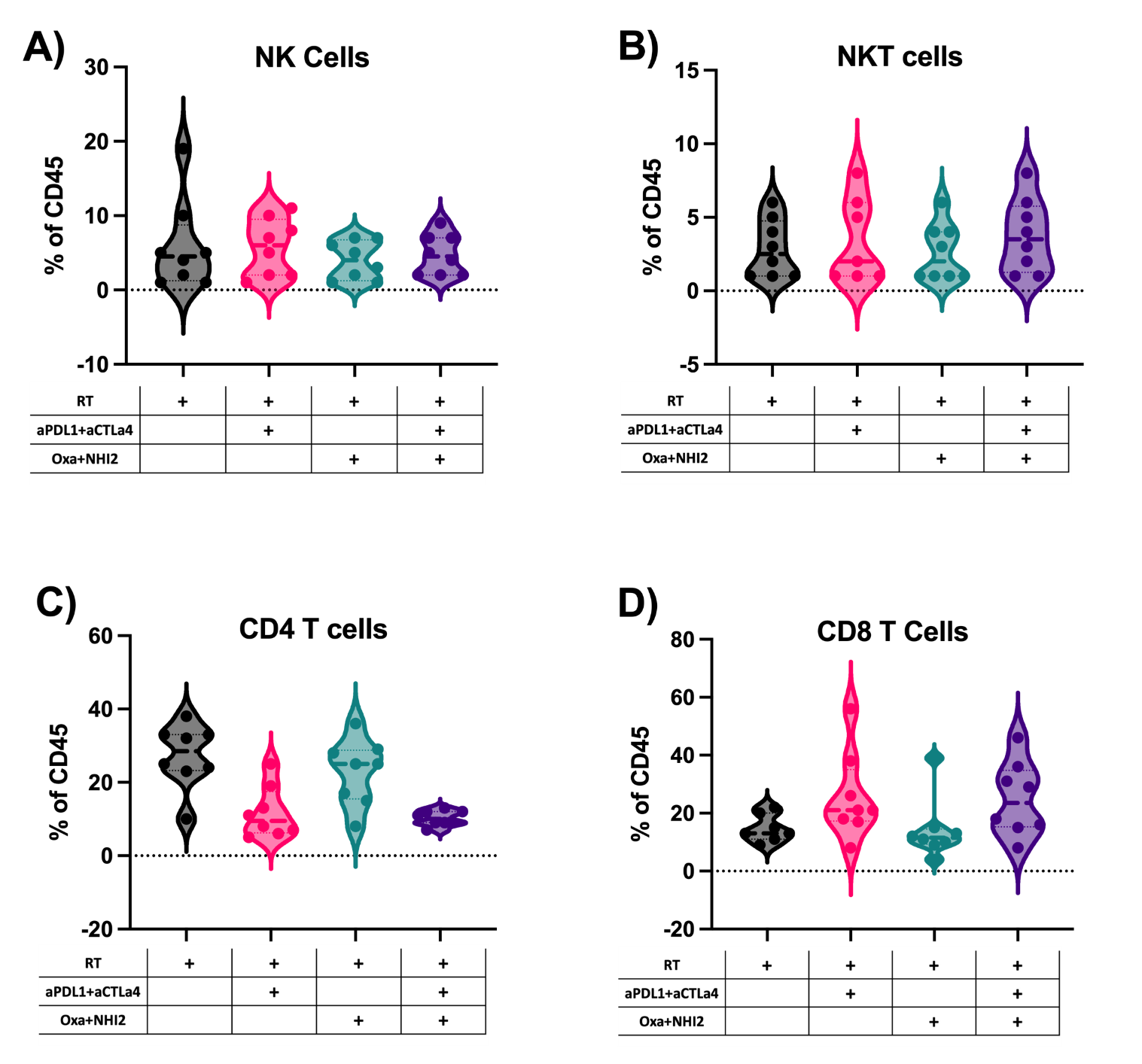


**Fig. S7. Lymphocytic cell profiling in tumor microenvironment following treatments.** Tumor-infiltrating NK, NKT and T cell populations were assessed for alterations in infiltration into the tumor microenvironment (TME) following RT, RT + ICIs, RT + LDHIs or RT + LDHIs + ICIs. There were no significant differences in the infiltration of (**A**) NK cells (CD45+/CD3-/NK1.1+); (**B**) NKT (CD45+/CD3+/NK1.1+); (**C**) CD4 T cells (CD45+/CD3+/CD4+/CD8-); or (**D)** CD8 T cells (CD45+/CD3+/CD8+/CD4-). Data represent the compilation of n=4 mice/group, repeated 2 separate times, for a total of 8 mice/group. One-way ANOVA tests with Tukey Post-Hoc test.

| Table S1-A | Total | Expected | Hits | Raw p | -log10(p) | Holm adjust | FDR | Impact |
| --- | --- | --- | --- | --- | --- | --- | --- | --- |
| Citrate cycle (TCA cycle) | 20 | 0.65 | 9 | 2.46E-09 | 8.61E+00 | 2.07E-07 | 2.07E-07 | 0.38 |
| Pyrimidine metabolism | 39 | 1.27 | 10 | 1.62E-07 | 6.79E+00 | 1.34E-05 | 6.79E-06 | 0.19 |
| Glyoxylate and dicarboxylate metabolism | 32 | 1.04 | 8 | 4.07E-06 | 5.39E+00 | 3.34E-04 | 1.14E-04 | 0.06 |
| Aminoacyl-tRNA biosynthesis | 48 | 1.56 | 8 | 9.81E-05 | 4.01E+00 | 7.95E-03 | 2.06E-03 | 0.00 |
| Alanine, aspartate, and glutamate metabolism | 28 | 0.91 | 6 | 1.90E-04 | 3.72E+00 | 1.52E-02 | 3.19E-03 | 0.34 |
| Arginine biosynthesis | 14 | 0.46 | 4 | 7.80E-04 | 3.11E+00 | 6.16E-02 | 1.09E-02 | 0.00 |
| Glycolysis / Gluconeogenesis | 26 | 0.85 | 5 | 1.16E-03 | 2.93E+00 | 9.08E-02 | 1.40E-02 | 0.22 |
| Valine, leucine, and isoleucine degradation | 40 | 1.30 | 6 | 1.44E-03 | 2.84E+00 | 1.11E-01 | 1.51E-02 | 0.12 |
| Pantothenate and CoA biosynthesis | 19 | 0.62 | 4 | 2.68E-03 | 2.57E+00 | 2.03E-01 | 2.50E-02 | 0.18 |
| Purine metabolism | 66 | 2.15 | 7 | 4.47E-03 | 2.35E+00 | 3.35E-01 | 3.59E-02 | 0.13 |
| Pyruvate metabolism | 22 | 0.72 | 4 | 4.70E-03 | 2.33E+00 | 3.48E-01 | 3.59E-02 | 0.19 |
| Propanoate metabolism | 23 | 0.75 | 4 | 5.55E-03 | 2.26E+00 | 4.06E-01 | 3.86E-02 | 0.32 |
| Phenylalanine, tyrosine, and tryptophan biosynthesis | 4 | 0.13 | 2 | 5.97E-03 | 2.22E+00 | 4.30E-01 | 3.86E-02 | 1.00 |
| Butanoate metabolism | 15 | 0.49 | 3 | 1.12E-02 | 1.95E+00 | 7.94E-01 | 5.98E-02 | 0.00 |
| Nicotinate and nicotinamide metabolism | 15 | 0.49 | 3 | 1.12E-02 | 1.95E+00 | 7.94E-01 | 5.98E-02 | 0.23 |
| Glutathione metabolism | 28 | 0.91 | 4 | 1.14E-02 | 1.94E+00 | 7.94E-01 | 5.98E-02 | 0.06 |
| Valine, leucine, and isoleucine biosynthesis | 8 | 0.26 | 2 | 2.56E-02 | 1.59E+00 | 1.00E+00 | 1.27E-01 | 0.00 |
| beta-Alanine metabolism | 21 | 0.68 | 3 | 2.85E-02 | 1.55E+00 | 1.00E+00 | 1.33E-01 | 0.06 |
| Phenylalanine metabolism | 12 | 0.39 | 2 | 5.56E-02 | 1.25E+00 | 1.00E+00 | 2.46E-01 | 0.36 |

| Table S1-B | Total | Expected | Hits | Raw p | -log10(p) | Holm adjust | FDR | Impact |
| --- | --- | --- | --- | --- | --- | --- | --- | --- |
| Citrate cycle (TCA cycle) | 20 | 0.56 | 8 | 1.81.E-08 | 7.74.E+00 | 1.52.E-06 | 9.09.E-07 | 0.35 |
| Aminoacyl-tRNA biosynthesis | 48 | 1.34 | 11 | 2.16.E-08 | 7.66.E+00 | 1.80.E-06 | 9.09.E-07 | 0.00 |
| Arginine biosynthesis | 14 | 0.39 | 6 | 8.31.E-07 | 6.08.E+00 | 6.82.E-05 | 2.33.E-05 | 0.08 |
| Alanine, aspartate, and glutamate metabolism | 28 | 0.78 | 7 | 6.02.E-06 | 5.22.E+00 | 4.87.E-04 | 1.26.E-04 | 0.39 |
| Glyoxylate and dicarboxylate metabolism | 32 | 0.89 | 6 | 1.72.E-04 | 3.76.E+00 | 1.38.E-02 | 2.90.E-03 | 0.06 |
| Pyrimidine metabolism | 39 | 1.09 | 6 | 5.37.E-04 | 3.27.E+00 | 4.24.E-02 | 6.77.E-03 | 0.04 |
| Glycolysis / Gluconeogenesis | 26 | 0.73 | 5 | 5.64.E-04 | 3.25.E+00 | 4.40.E-02 | 6.77.E-03 | 0.22 |
| Pyruvate metabolism | 22 | 0.61 | 4 | 2.66.E-03 | 2.58.E+00 | 2.05.E-01 | 2.79.E-02 | 0.19 |
| Phenylalanine, tyrosine, and tryptophan biosynthesis | 4 | 0.11 | 2 | 4.40.E-03 | 2.36.E+00 | 3.34.E-01 | 4.11.E-02 | 1.00 |
| Butanoate metabolism | 15 | 0.42 | 3 | 7.27.E-03 | 2.14.E+00 | 5.45.E-01 | 6.11.E-02 | 0.00 |
| D-Glutamine and D-glutamate metabolism | 6 | 0.17 | 2 | 1.06.E-02 | 1.97.E+00 | 7.85.E-01 | 8.10.E-02 | 0.00 |
| Valine, leucine, and isoleucine biosynthesis | 8 | 0.22 | 2 | 1.91.E-02 | 1.72.E+00 | 1.00.E+00 | 1.25.E-01 | 0.00 |
| Arginine and proline metabolism | 38 | 1.06 | 4 | 1.94.E-02 | 1.71.E+00 | 1.00.E+00 | 1.25.E-01 | 0.15 |
| Glutathione metabolism | 28 | 0.78 | 3 | 4.07.E-02 | 1.39.E+00 | 1.00.E+00 | 2.35.E-01 | 0.05 |
| Phenylalanine metabolism | 12 | 0.33 | 2 | 4.20.E-02 | 1.38.E+00 | 1.00.E+00 | 2.35.E-01 | 0.36 |
| Inositol phosphate metabolism | 30 | 0.84 | 3 | 4.86.E-02 | 1.31.E+00 | 1.00.E+00 | 2.55.E-01 | 0.13 |
| Nicotinate and nicotinamide metabolism | 15 | 0.42 | 2 | 6.34.E-02 | 1.20.E+00 | 1.00.E+00 | 3.13.E-01 | 0.23 |

| Table S1-C | Total | Expected | Hits | Raw p | -log10(p) | Holm adjust | FDR | Impact |
| --- | --- | --- | --- | --- | --- | --- | --- | --- |
| Citrate cycle (TCA cycle) | 20 | 0.72 | 10 | 2.10E-10 | 9.68E+00 | 1.76E-08 | 1.76E-08 | 0.44 |
| Aminoacyl-tRNA biosynthesis | 48 | 1.72 | 13 | 2.75E-09 | 8.56E+00 | 2.28E-07 | 1.15E-07 | 0.00 |
| Arginine biosynthesis | 14 | 0.50 | 8 | 4.11E-09 | 8.39E+00 | 3.37E-07 | 1.15E-07 | 0.19 |
| Alanine, aspartate, and glutamate metabolism | 28 | 1.00 | 9 | 2.03E-07 | 6.69E+00 | 1.65E-05 | 4.27E-06 | 0.58 |
| Glyoxylate and dicarboxylate metabolism | 32 | 1.15 | 8 | 8.73E-06 | 5.06E+00 | 6.98E-04 | 0.00014664 | 0.06 |
| Pyrimidine metabolism | 39 | 1.40 | 8 | 4.20E-05 | 4.38E+00 | 3.32E-03 | 0.0005882 | 0.10 |
| Butanoate metabolism | 15 | 0.54 | 5 | 1.12E-04 | 3.95E+00 | 8.77E-03 | 0.0013492 | 0.00 |
| Glycolysis / Gluconeogenesis | 26 | 0.93 | 6 | 2.13E-04 | 3.67E+00 | 1.64E-02 | 0.0022318 | 0.22 |
| D-Glutamine and D-glutamate metabolism | 6 | 0.22 | 3 | 8.08E-04 | 3.09E+00 | 6.14E-02 | 0.0068324 | 0.50 |
| Pyruvate metabolism | 22 | 0.79 | 5 | 8.13E-04 | 3.09E+00 | 6.14E-02 | 0.0068324 | 0.19 |
| Valine, leucine, and isoleucine biosynthesis | 8 | 0.29 | 3 | 2.15E-03 | 2.67E+00 | 1.59E-01 | 0.016417 | 0.00 |
| Glutathione metabolism | 28 | 1.00 | 5 | 2.58E-03 | 2.59E+00 | 1.88E-01 | 0.018025 | 0.08 |
| Pantothenate and CoA biosynthesis | 19 | 0.68 | 4 | 3.84E-03 | 2.42E+00 | 2.76E-01 | 0.024794 | 0.18 |
| Phenylalanine, tyrosine, and tryptophan biosynthesis | 4 | 0.14 | 2 | 7.23E-03 | 2.14E+00 | 5.13E-01 | 0.043389 | 1.00 |
| Arginine and proline metabolism | 38 | 1.36 | 5 | 1.00E-02 | 2.00E+00 | 7.00E-01 | 0.056034 | 0.23 |
| Valine, leucine, and isoleucine degradation | 40 | 1.43 | 5 | 1.24E-02 | 1.91E+00 | 8.57E-01 | 0.065211 | 0.03 |
| Nicotinate and nicotinamide metabolism | 15 | 0.54 | 3 | 1.46E-02 | 1.84E+00 | 9.94E-01 | 0.072194 | 0.23 |
| Nitrogen metabolism | 6 | 0.22 | 2 | 1.73E-02 | 1.76E+00 | 1.00E+00 | 0.080547 | 0.00 |
| Purine metabolism | 66 | 2.37 | 6 | 2.77E-02 | 1.56E+00 | 1.00E+00 | 0.1225 | 0.10 |
| Propanoate metabolism | 23 | 0.82 | 3 | 4.65E-02 | 1.33E+00 | 1.00E+00 | 0.19517 | 0.05 |

**Table S1. Metabolomic data analysis with MetaboAnalyst 5.0.** The Pathway Analysis module of MetaboAnalyst 5.0 combines results from powerful pathway enrichment analysis with pathway topology analysis to identify the most relevant pathways involved in the conditions under study. It uses high-quality KEGG metabolic pathways as the backend knowledgebase. This module integrates many well-established (i.e., univariate analysis, over-representation analysis) methods, as well as novel algorithms and concepts (i.e. Global Test, GlobalAncova, network topology analysis) into pathway analysis.

The tables below show the detailed results from the pathway analysis for (**A**) 15 mM Oxamate, (**B**) 18 μM NHI-2, and (**C**) combination treatment groups. The statistical *P* values from enrichment analysis were further adjusted for multiple testing. In particular, the **Total** is the total number of compounds in the pathway; the **Hits** is the actually matched number from the user uploaded data; the **Raw** **p** is the original *P* value calculated from the enrichment analysis; the **Holm p** is the *P* value adjusted by Holm-Bonferroni method; the **FDR p** is the *P* value adjusted using False Discovery Rate; the **Impact** is the pathway impact value calculated from pathway topology analysis.
